# Heart atlas for retrospective cardiac dosimetry: a multi-institutional study on interobserver contouring variations and their dosimetric impact

**DOI:** 10.1186/s13014-021-01965-5

**Published:** 2021-12-20

**Authors:** Marcus Stockinger, Heiko Karle, Hannes Rennau, Sabine Sebb, Ulrich Wolf, Julia Remmele, Sandra Bührdel, Detlef Bartkowiak, Maria Blettner, Heinz Schmidberger, Daniel Wollschläger

**Affiliations:** 1 Department of Radiation Oncology and Radiation Therapy, University Medical Center of the Johannes Gutenberg-University Mainz, Langenbeckstr. 1, 55131 Mainz, Germany; 2 Department of Radiation Oncology, University Hospital Rostock, Südring 75, 18059 Rostock, Germany; 3 Department of Radiation Oncology, University Hospital Leipzig, Stephanstraße 9a, 04103 Leipzig, Germany; 4 Department of Radiation Oncology, University Hospital Ulm, Albert-Einstein-Allee 23, 89081 Ulm, Germany; 5 Institute of Medical Biostatistics, Epidemiology and Informatics (IMBEI), University Medical Center of the Johannes Gutenberg-University Mainz, Obere Zahlbacher Str. 69, 55131 Mainz, Germany

**Keywords:** Atlas, Heart dose, Contouring, Retrospective dosimetry

## Abstract

**Purpose:**

Cardiac effects after breast cancer radiation therapy potentially affect more patients as survival improves. The heart’s heterogeneous radiation exposure and composition of functional structures call for establishing individual relationships between structure dose and specific late effects. However, valid dosimetry requires reliable contouring which is challenging for small volumes based on older, lower-quality computed tomography imaging. We developed a heart atlas for robust heart contouring in retrospective epidemiologic studies.

**Methods and materials:**

The atlas defined the complete heart and geometric surrogate volumes for six cardiac structures: aortic valve, pulmonary valve, all deeper structures combined, myocardium, left anterior myocardium, and right anterior myocardium. We collected treatment planning records from 16 patients from 4 hospitals including dose calculations for 3D conformal tangential field radiation therapy for left-sided breast cancer. Six observers each contoured all patients. We assessed spatial contouring agreement and corresponding dosimetric variability.

**Results:**

Contouring agreement for the complete heart was high with a mean Jaccard similarity coefficient (JSC) of 89%, a volume coefficient of variation (CV) of 5.2%, and a mean dose CV of 4.2%. The left (right) anterior myocardium had acceptable agreement with 63% (58%) JSC, 9.8% (11.5%) volume CV, and 11.9% (8.0%) mean dose CV. Dosimetric agreement for the deep structures and aortic valve was good despite higher spatial variation. Low spatial agreement for the pulmonary valve translated to poor dosimetric agreement.

**Conclusions:**

For the purpose of retrospective dosimetry based on older imaging, geometric surrogate volumes for cardiac organs at risk can yield better contouring agreement than anatomical definitions, but retain limitations for small structures like the pulmonary valve.

**Supplementary Information:**

The online version contains supplementary material available at 10.1186/s13014-021-01965-5.

## Introduction

For women, breast cancer remains the most frequently diagnosed cancer and the leading cause of cancer-related death [1]. Adjuvant radiation therapy (RT) was shown to reduce mortality and local recurrence [2], among other treatment advances like targeted immunotherapy and endocrine therapy. Correspondingly, treatment outcome has improved with ten-year relative survival in some countries now surpassing 80% [3].

Better survival mandates a focus on long-term adverse treatment effects which can affect more patients. Aside from potentially cardiotoxic chemotherapy and anti-HER2 immunotherapy [4], breast cancer RT can expose the heart to high levels of radiation [5, 6]. The need to address late cardiac effects of breast cancer treatment is now widely recognized [4, 7]. Specifically, radiation dose to the heart was repeatedly shown to be linked to late cardiac disease [8–11] with mixed evidence for more recent treatment years [12–14]. Radiation therapy-induced late cardiac effects are also relevant for patients with Hodgkin lymphoma [15], esophageal cancer [16], and potentially for lung cancer patients [17, 18] with improved survival due to immune-checkpoint inhibitor treatment.

In retrospective epidemiologic studies on long term cardiac risks after radiation therapy [8, 9], accurate and reliable heart dosimetry is a prerequisite for valid dose–response analysis. However, the heart’s proximity to the steep dose gradient at the field border in breast cancer RT leads to a heterogeneous dose distribution [19], rendering mean heart dose less representative [20]. Doses for functional substructures are thus desirable as predictors for specific cardiac endpoints like myocardial infarction [20, 21]. In particular, the left anterior descending coronary artery (LAD) has potentially high radiation exposure in left-sided breast cancer RT [22], and frequently is the location of coronary artery stenosis [23]. Measures for high exposure like the relative volume exposed to at least 5 Gy [9], or the dose to the most exposed 2 cm^3^ [24] have therefore been proposed as risk factors.

In retrospective epidemiologic studies, image quality from older computed tomography (CT) scans may be limited, without contrast enhancement, and with poor spatial resolution. Due to poor visibility, contouring relevant anatomical substructures thus becomes difficult in the context of such studies. This prevents using heart atlases that are well established for clinical treatment planning which define detailed anatomical features useful for setting dose constraints in individual patients. Unlike in the clinical setting, the restricted quality of CT imaging material available to retrospective epidemiologic studies usually rules out fine-grained contouring, such as of individual segments of the left ventricle or LAD [31–35].

Combined with the heterogeneous dose distribution, contour segmentation uncertainty can substantially contribute to heart dose measurement error [25–31]. Previously, Tan [36] suggested the *Anterior Myocardial Territory* as a surrogate organ at risk that contains the LAD. We here extend and refine this approach to a complete heart atlas.

For a retrospective cohort study on late cardiac effects after breast cancer RT [37], we developed a contouring atlas for the complete heart and functional substructures as potential organs at risk [19]. Substructure definitions aimed to ensure sufficient contouring reliability even with limited CT image quality, low spatial resolution, and poor visibility of the coronary artery. In a multi-institutional validation study, we quantified the structures’ spatial and dosimetric uncertainty caused by contouring variations.

## Methods and materials

### Patients and observers

Sixteen patients with breast cancer RT from 1998 to 2014 were selected from 4 university medical centers (C1, C2, C3, C4) together with CT scans and initial dose calculations. Patients with pectus excavatum were ineligible. The patients were part of two different dosimetry samples from the PASSOS project on late cardiac effects after breast cancer therapy [19, 37–39].

All patients were contoured by 6 observers (3 medical physicists, 2 technologists, 1 physician) from the 4 participating hospitals. Observers had prior experience with the heart atlas described below but had no shared training on its use. Instead, they relied on written instructions. Observers delineated the contours independently without communicating. Since all observers had a similar level of expertise, none was designated as gold standard.

### Heart atlas

We developed a heart atlas with standard operating procedures for segmentation of the complete heart, and for 6 cardiac substructures as potential organs at risk for late cardiac effects. The heart atlas was developed as the consensus definition of an expert group that reviewed high resolution chest CT scans with contrast enhancement to determine suitable surrogate volumes for anatomical structures. The detailed illustrated contouring instructions are provided as an additional file [see Additional file 1].

**Fig. 1:**
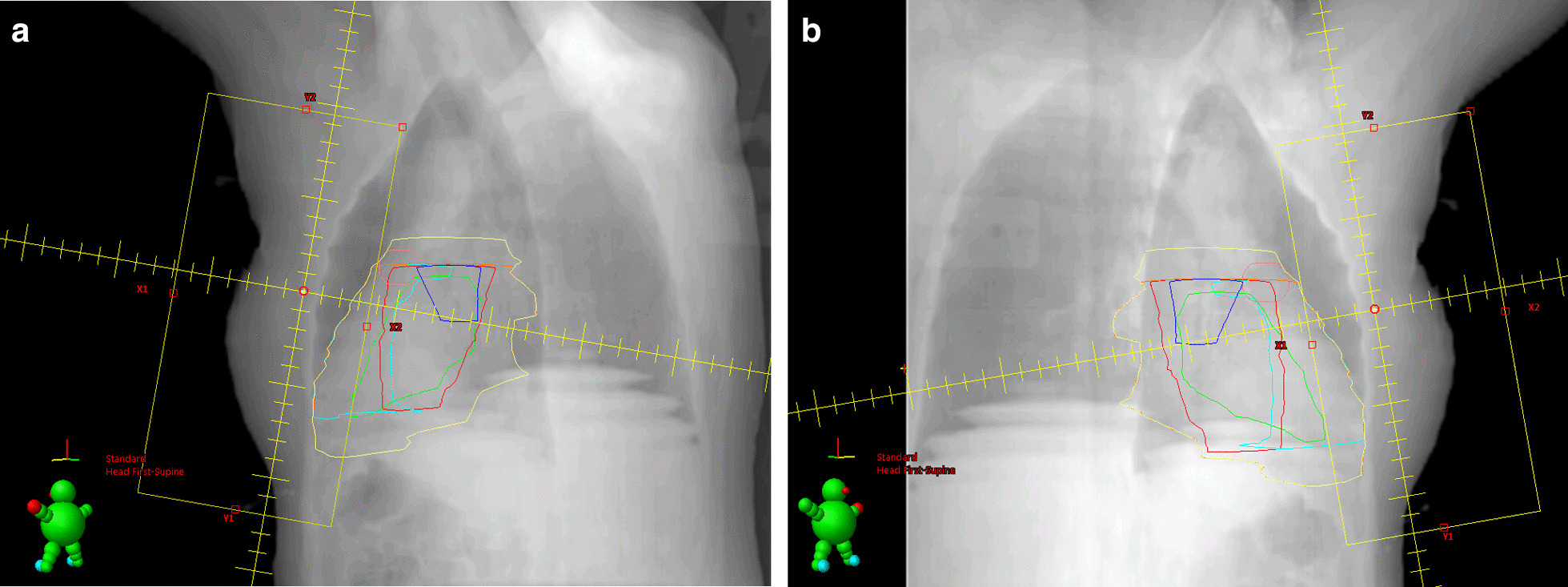
Heart atlas. Sagittal CT slices (**a**: patient facing left,** b**: patient facing right) with superimposed contours of the complete heart (orange) and functional substructures as defined by the study protocol: Aortic valve (light pink), Pulmonary valve (blue), deep structures (light green), right anterior myocardium (light blue), left anterior myocardium (red), and complete myocardium (purple region)

The substructures were defined as geometrical surrogate volumes of anatomical structures (Fig. 1):

- The complete heart according to the Radiation Therapy Oncology Group definition for RT in breast cancer [40].
- The aortic valve: A conical volume starting at the aortic root going 3 cm in the caudal direction.
- The pulmonary valve: A spherical volume expanding uniformly around a point within the truncus pulmonalis at the level of the aortic root until it almost touches the aortic valve volume.
- The heart wall including the pericardium, coronary arteries, and the myocardium without the ventricular septum: The outer layer of the complete heart below the aortic valve with a thickness of 1 cm.
- The left anterior heart wall including the left LAD.
- The right anterior heart wall including the right coronary artery and the sinoatrial node.
- A surrogate volume to summarily capture dose to the deep cardiac structures like the atrioventricular node, bundle of His, Septum, and mitral valve: A 2 cm symmetrical inner (negative) margin applied to the complete heart.

### Dosimetry

Treatment planning was done for left-sided breast cancer in the contributing hospital. Planning target volume was between 650 and 1550 ml (mean 1050 ml). The plan for all patients comprised one pair of 6 MV tangential fields and hard wedges with a total PTV dose of 50 Gy. For the boost series, electron (5 patients) and photon (11 patients) plans with a dose of 10 Gy were implemented. Lymph node irradiation fields were not investigated.

Dose calculation for photon beams in C1 and C2 was performed using Varian’s anisotropic analytical algorithm (AAA) [41]. Electron beams were calculated using Varian’s electron Monte Carlo algorithm [42]. For both algorithms, a 2.5 mm dose grid was used. Dose calculations in C3 and C4 were carried out using Oncentra Masterplan version 4.3 and its Collapsed Cone algorithm with a dose grid of 3 mm [43].

CT scans were done without contrast enhancement and with slice thickness 10 mm (3 patients), 7.5 mm (4 patients), 5 mm (1 patient), or 3 mm (8 patients). DICOM imaging files were distributed to all participating observers. After contouring, files were collected in C1, imported into Varian Eclipse V15.1, and included in a single integrated structure set for each patient.

Each contoured structure was exported as a 3D surface triangle mesh using the Eclipse Scripting API [44]. Its cumulative dose volume histogram (DVH) was exported with a bin size of 10 cGy. The DVHs were imported into the statistical environment R [45, 46]. For all structures, we calculated the volume-weighted mean dose (DMEAN), the dose received by the maximally exposed 2 cm^3^ (D2CC), and the relative volume with a dose of at least 5 Gy (V5GY).

### Statistical agreement measures

Differences between individual contours along several directions were qualitatively explored by measuring distances within the treatment planning system. Spatial contouring agreement was systematically evaluated from pairwise similarity of structure delineations using distance measures, volume overlap, and volume variation [47–49]. Detailed illustration on the calculation steps for the following measures are provided as an additional file [see Additional file 2].

We determined union and intersection volumes of the structures﻿’ 3D surface meshes for the 15 observer pairs [50]. Volume overlap was calculated using the Dice similarity coefficient (DSC, twice the intersection volume divided by the sum of both volumes) as well as the Jaccard similarity coefficient (JSC, intersection volume divided by union volume).

For each structure, several distance-based agreement measures for the 15 observer pairs were calculated: the Euclidean distance of the respective centers of mass (DCOM), the average surface distance (ASD), and the Hausdorff distance (HD) between the two meshes. ASD and HD are based on the set of vertex-by-vertex Euclidean distances between the two meshes with ASD being the average, and HD being the maximum [48]. Overall pairwise spatial agreement for one structure as assessed by DSC, JSC, DCOM, ASD, and HD, was determined by averaging values over observer pairs and patients.

Variability in structure volume was assessed with the coefficient of variation (CV), i.e., the ratio of the standard deviation to the mean. The CV was derived from the structure-specific error variances in a log-normal regression model with a covariate that indexed all combinations of structure and patient. Additionally, the intraclass correlation coefficient (ICC(2)) was calculated as a measure of structure volume agreement [51].

The dosimetric impact of spatial contouring discrepancies for the given dose distribution was quantified with the CV for DMEAN and D2CC as for structure volume. For V5GY, the standard deviation (SD) was derived from the structure-specific mean and precision parameters in a beta regression with a covariate that indexed all combinations of structure and patient. In addition, ICC(2) for DMEAN, D2CC, and V5GY was calculated as for structure volume.

## Results

### Qualitative assessment

**Fig. 2:**
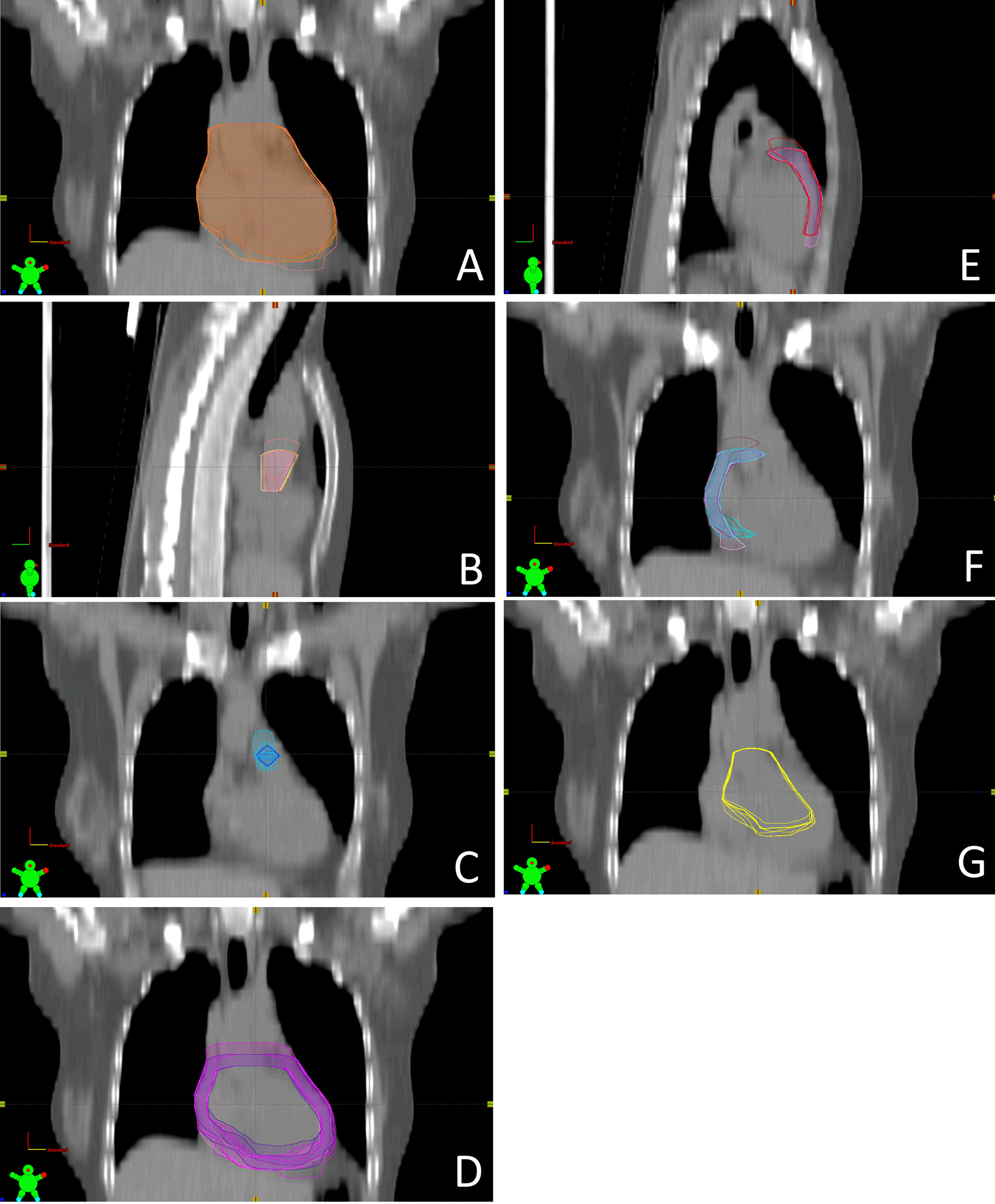
Variations in delineated structure contours among observers illustrated in a sample of CT slices. **A** Complete heart. **B** Aortic valve. **C** Pulmonary valve. **D** Myocardium. **E** Right anterior myocardium. **F** Left anterior myocardium. **G** Deep structures

The complete heart could be demarcated with deviations typically below 3 mm. Larger deviations could be identified on the right heart base, the posterior wall of the left atrium, and the right hilum in the axial planes. Deviations in these regions could reach 5 mm in the axial plane and 10 mm in the cranio-caudal direction (Fig. 2A).

For the pulmonary valve, differences up to 15 mm occurred in the cranio-caudal direction as well as in the medio-lateral direction (Fig. 2B). The aortic valve also showed deviations of up to 15 mm in the cranio-caudal direction and about 10 mm laterally (Fig. 2C). The myocardium volume exhibited deviations of up to 15 mm at its cranial end (Fig. 2D), especially in the region of the heart base and the hilum, as well as near the posterior wall of the left atrium. The largest deviations in the deep structures volume were seen in the caudal and ventral area like in the complete heart (Fig. 2G).

### Quantitative spatial agreement

**Fig. 3:**
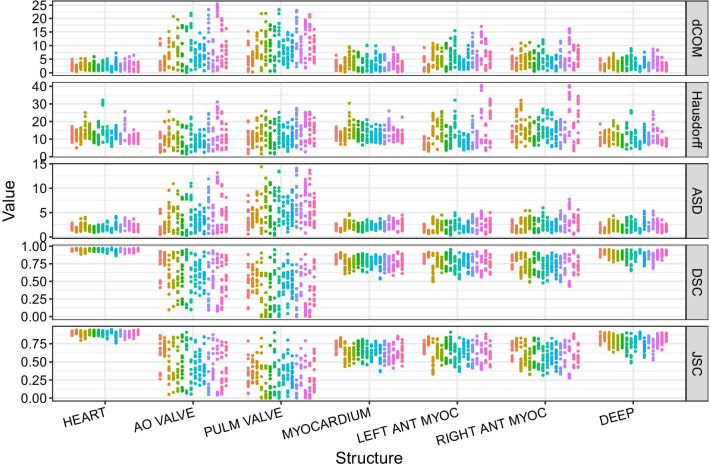
Spatial contouring agreement measures for each heart atlas structure. Each point represents the similarity value for one observer pair for one patient (color coded) and structure. Distance-based measures: distance of of the centers of mass (DCOM), Hausdorff distance, average surface distance (ASD). Volume-overlap measures: Dice similarity coefficient (DSC), Jaccard similarity coefficient (JSC)

Pairwise volume overlap for the complete heart amounted to 89.2% average JSC. Substructures had lower average JSC values, with smaller substructures exhibiting worse overlap than larger substructures (Table 1, Fig. 3). Here, JSC values ranged from 78.0% (deep structures) to 28.5% (pulmonary valve).

**Table 1:** Volume for each heart atlas structure averaged over patients and observers

Structure	M volume [mm^3^]	CV volume [%]	ICC volume	DCOM [mm]	ASD [mm]	HD [mm]	JSC	DSC
Heart	693.2	5.24 (4.52–6.20)	0.83 (0.62–0.93)	2.27	2.01	12.7	0.89	0.94
Aortic valve	13.6	21.3 (18.9–25.0)	0.53 (0.32–0.76)	7.96	4.16	10.8	0.44	0.57
Pulmonary valve	8.34	69.5 (58.1–84.7)	0.50 (0.27–0.74)	9.47	5.65	12.7	0.28	0.40
Myocardium	344.1	5.74 (4.96–6.75)	0.75 (0.55–0.89)	3.25	2.28	13.3	0.64	0.78
Left anterior myocardium	67.6	9.78 (8.41–11.5)	0.75 (0.58–0.88)	5.28	2.27	13.0	0.63	0.76
Right anterior myocardium	57.9	11.5 (9.87–13.5)	0.59 (0.38–0.79)	5.03	2.75	16.1	0.58	0.72
Deep structures	115.7	13.9 (12.0–16.4)	0.70 (0.44–0.87)	3.03	2.15	11.0	0.78	0.88

Spatial contouring agreement measures for each heart atlas structure. CV: coefficient of variation (with 95% credible interval). ICC: Intraclass correlation coefficient (with 95% credible interval). DCOM: Distance of center of mass. ASD: Average surface distance. HD: Hausdorff distance. JSC: Jaccard similarity coefficient. DSC: Dice similarity coefficient

Pairwise distance measures showed the same pattern with the shortest average DCOM of 2.3 mm for the complete heart, and larger DCOM values in substructures, ranging from 3.0 mm (deep structures) to 9.5 mm (pulmonary valve). Likewise, ASD was lowest for the complete heart at 2.0 mm with substructures ranging from 2.2 mm (deep structures) to 5.7 mm (pulmonary valve). Pairwise Hausdorff distances behaved differently with 12.7 mm for the complete heart and a range from 10.8 mm (aortic valve) to 16.1 mm (right anterior myocardium).

**Fig. 4:**
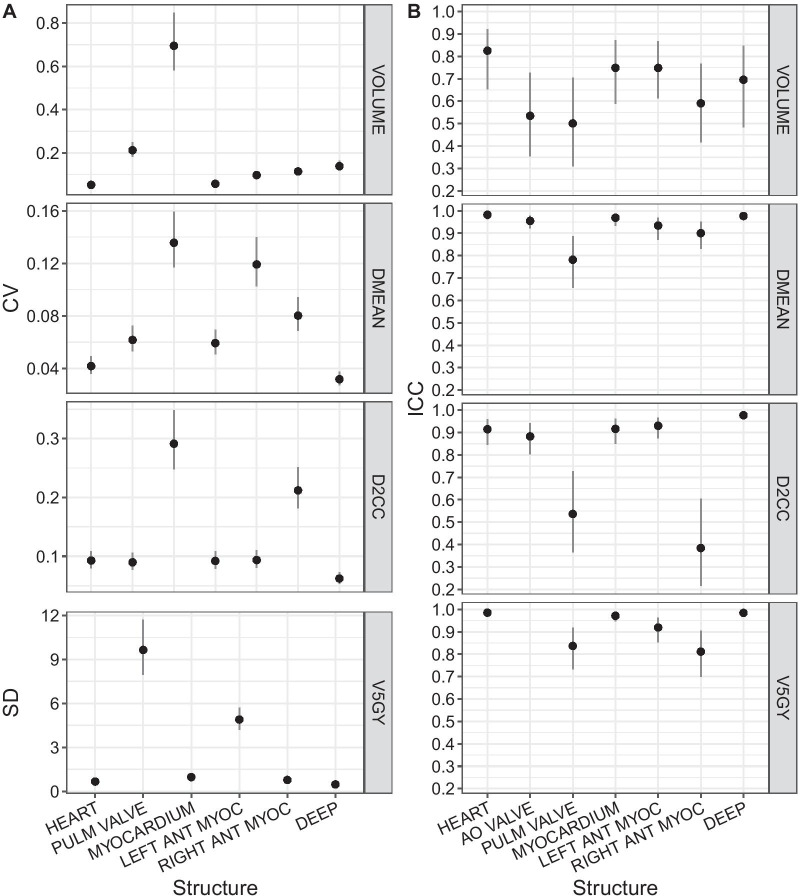
Contouring agreement measures. **A** Coefficient of variation (CV) with 95% credible interval for all structures for D2CC, DMEAN, and structure volume. Standard deviation (SD) for V5GY with 95% credible interval. **B** Intraclass correlation coefficient (ICC) with 95% confidence interval for all structures for D2CC, DMEAN, V5GY, and structure volume

Variations in volume were smallest for the complete heart with a CV of 5.2%, and higher for substructures, ranging from 5.7% (myocardium) to 69.5% (pulmonary valve) (Table 2, Fig. 4). Correspondingly, observer agreement was highest for the complete heart volume with an ICC value of 0.83, ranging from 0.75 (myocardium) to 0.50 (pulmonary valve).

**Table 2:** Mean dose (DMEAN), extreme dose (D2CC), and relative volume with a dose of at least 5 Gy (V5GY) for each heart atlas structure averaged over patients and observers

Structure	M DMEAN [Gy]	M D2CC [Gy]	M V5GY [%]	CV DMEAN [%]	CV D2CC [%]	SD V5GY [% points]	ICC DMEAN	ICC D2CC	ICC V5GY [%]
Heart	3.97	43.8	13.1	4.18 (3.59–4.93)	9.28 (7.99–10.8)	0.67 (0.57–0.78)	0.98 (0.96–0.99)	0.91 (0.83–0.97)	0.99 (0.97–0.99)
Aortic valve	1.62	1.85	0.00	6.17 (5.30–7.24)	8.97 (7.70–10.6)		0.95 (0.91–0.98)	0.88 (0.78–0.95)	
Pulmonary valve	3.79	4.83	16.2	13.6 (11.7–15.9)	29.1 (24.8–34.8)	9.64 (7.96–11.7)	0.78 (0.63–0.90)	0.54 (0.33–0.76)	0.84 (0.71–0.93)
Myocardium	5.15	43.8	18.9	5.92 (5.10–6.95)	9.20 (7.90–10.8)	0.97 (0.83–1.13)	0.97 (0.92–0.99)	0.92 (0.83–0.97)	0.97 (0.94–0.99)
Left anterior myocardium	13.5	42.2	61.3	11.9 (10.3–14.0)	9.36 (8.05–11.0)	4.90 (4.20–5.70)	0.93 (0.85–0.97)	0.93 (0.86–0.97)	0.92 (0.84–0.97)
Right anterior myocardium	1.74	3.56	1.07	8.02 (6.89–9.43)	21.2 (18.2–25.1)	0.77 (0.63–0.96)	0.90 (0.81–0.96)	0.38 (0.19–0.64)	0.81 (0.67–0.92)
Deep structures	2.36	6.58	2.60	3.18 (2.74–3.74)	6.22 (5.35–7.31)	0.47 (0.40–0.55)	0.99 (0.97–0.99)	0.98 (0.96–0.99)	0.98 (0.97–0.99)

Dose-based contouring agreement measures for all heart atlas structures with 95% credible interval. CV: coefficient of variation. ICC: Intraclass correlation coefficient. SD: Standard deviation. DMEAN: Volume weighted mean dose. D2CC: Dose to the maximally exposed 2 cm^3^. V5GY: Relative volume with a dose of at least 5 Gy. V5GY for the aortic valve was always 0, SD and ICC were therefore not calculated

### Quantitative dosimetric agreement

Impact of spatial contouring variations on absorbed dose variability was quantified as a CV of 4.2% (9.3%) for DMEAN (D2CC) of the complete heart (Table 2). Substructures showed a range from 3.2% (6.2%) for DMEAN (D2CC) in the deep structures to 13.6% (29.1%) for DMEAN (D2CC) in the pulmonary valve. SD for V5GY was 0.67 percentage points in the complete heart and ranged between 0.47 percentage points in the deep structures and 9.6 percentage points in the pulmonary valve.

A similar pattern was obtained for ICC. In the complete heart, values were 0.98 for DMEAN, 0.91 for D2CC, and 0.99 for V5GY. ICC in substructures was highest for the deep structures with values of 0.99 for DMEAN, 0.98 for D2CC, and 0.98 for V5GY. ICC for DMEAN was lowest in the pulmonary valve (0.78), while ICC for D2CC (0.38) and V5GY (0.81) was lowest in the right anterior myocardium.

## Discussion

For retrospective cardiac dosimetry, we developed a heart atlas to define surrogate volumes for functional structures that could be reliably contoured in CT scans of limited quality. In a multi-institutional study, we analyzed inter-observer contouring agreement with respect to structure geometry and dose.

Agreement for the complete heart was high, both spatially as well as with respect to dose. Myocardium-based structures showed fair spatial agreement while delineations for the valves differed considerably in location. The large observed deviations for the pulmonary valve and aortic valve were probably due to limited presentation or visibility of the coronary artery, their starting point for contouring. Since the pulmonary valve was close to the field border, its low spatial agreement translated into poor dosimetric agreement. Despite spatial variations, dosimetric agreement was fair for the aortic valve which was further away from the dose gradient. Except for high dose measures in the right anterior myocardium, other dose metrics showed good to excellent agreement. The V5GY metric proposed as a predictor for cardiac risk [9] showed good reliability for the complete heart, and for myocardium-based structures.

There was a direct correspondence between structure volume and spatial similarity with the complete heart consistently being the most similar on all measures, and the pulmonary valve being the least similar. However, the Hausdorff distance as a measure for extreme instead of average spatial separation provided an exception from this pattern.

### Strengths and limitations

The heart atlas captures cardiac organs at risk that are relevant in retrospective epidemiologic studies on late cardiac effects after radiation therapy for different cancer entities with heterogeneous dose distribution in the heart. In particular, the volume of the left anterior myocardium typically contains important anatomical structures like the LAD, making it an important organ at risk [36]. In contrast to other suggestions for surrogate volumes to organs at risk [21, 36], the structures suggested here can be contoured even when coronary arteries are poorly visible. While our atlas is not intended for clinical treatment planning, it enables retrospective epidemiologic studies to use a maximum amount of available imaging data for dose–response analyses in larger patient groups.

By using CT scans without contrast enhancement and with slice thickness up to 10 mm, our validation results are based on realistic data for retrospective cohort studies on patients with treatment around the year 2000. Patient and observer selection from 4 independent hospitals reflect typical study scenarios and thus supports the generalizability of our validation results. As retrospective dosimetry is often handled by medical physics staff instead of clinicians, the sample of observers is realistic for epidemiologic studies.

We provide a comprehensive reliability assessment of our heart atlas including distance-based and volume-based spatial contouring agreement measures [48]. Dose-based agreement includes several measures for dose metrics that are candidate predictors for late cardiac effects.

Several limitations apply to the results. The atlas was motivated by the problem that small anatomical structures are not reliably visible in low resolution CT scans without contrast enhancement typically available in retrospective epidemiologic studies. However, this implies that there is uncertainty whether the defined surrogate volumes indeed perfectly enclose the targeted anatomical structures. As observers in this study were aware that their contouring was assessed, and hence may have been more conscientious, our results may be too optimistic compared to contouring during daily routine. As no gold standard was defined, we cannot rule out systematic bias in contouring that was shared among all participating observers.

Since the dosimetric evaluation of observer agreement depends on the radiation fields, our results for dose-based agreement are specific to 3D conformal tangential breast cancer RT for left-sided tumors which lead to higher heart dose variability than right-sided tumors.

### Comparison to previous heart atlas validation studies

Previous studies using the same indicators for interobserver variability also showed high contouring reliability for the complete heart. Li et al. [27] reported 0.86 JSC, 8 mm DCOM, and 3.8 mm ASD where we obtained 0.89 JSC, 0.94 DSC, 2.3 mm DCOM, and 2.0 mm ASD. Results from Feng et al. [26] were 0.89 JSC, Nielsen et al. [29] reported 0.94 DSC, Groom et al. [30] obtained 0.95 DSC, Lorenzen et al. [28] published 0.88 JSC and 0.93 DSC. With respect to dose variations, Lorenzen et al. [28] calculated a CV of 3.6% for mean dose and 4.0% for maximum dose compared to a CV of 4.2% for mean dose and 9.3% for D2CC in our study.

Feng et al. [26] calculated spatial agreement for the LAD and obtained 0.34 JSC, much lower than 0.63 in the surrogate volume of the left anterior myocardium in our study. Like Nielsen et al. [29], Lorenzen et al. [28] did not calculate spatial agreement for the LAD due to its small volume. Their reported dose variability of 29% CV for mean dose, and 31% for maximum dose of the LAD is higher than our results of 12% for mean dose and 9.4% for D2CC in the left anterior myocardium.

Duane et al. [31] presented results on the contouring reliability for coronary arteries as defined by their newly-developed atlas. With DSC values ranging between 0.10 and 0.53 for coronary arterial segments, overlap was small even with a CT slice thickness of 3 mm. Nevertheless, Hausdorff distances were only between 1.3 and 5.1 mm. For LAD segments near a high dose gradient, the small spatial displacement translated into differences in absorbed dose between about 20 Gy and 40 Gy for a partially wide tangential technique. Based on 3 raters from the same department who delineated the main coronary arteries, Wennstig et al. [33] reported good concordance for mean dose as quantified by ICC, but did not report volume overlap measures.

Based on contrast-enhanced CT-angiographies, Munshi et al. [52] proposed a “coronary strip” as a new organ at risk representing the left and the right coronary artery. However, there was considerable variation among the 51 analyzed hearts. For large dosimetric studies, a generous definition like left and right anterior wall may cover the anatomical range of critical structures more reliably, also with respect to organ movement.

### Implications for the analysis of dose–response relationships

Not taking into account uncertainty of dose estimates can make dose–response models for radiation-induced late effects biased and less efficient [30, 53]. By quantifying the uncertainty behind individual dose estimates in the model, it is possible to reduce this bias and increase efficiency.

Consistent with previous studies [29], we found that uncertainty for mean dose and extreme dose in the complete heart is low, with a CV of about 5%. While uncertainty for substrate volumes of the left and right anterior myocardium is higher, reliability is still better than previously reported agreement for contouring the LAD and right coronary artery directly [26–28]. This suggests the possibility of using these surrogate structures in specific dose–response analyses for diseases related to heart vascularization.

In contrast, there was considerable spatial uncertainty associated with delineating the valves with the given CT imaging data. Since the pulmonary valve also shows poor dosimetric reliability, this result limits the possibility to establish specific dose–response models for associated late effects like valvular dysfunction.

Dose–response analyses also need to consider other sources of measurement error. These include patient positioning and immobilization [54], organ movement [55], and reduced out-of-field dose calculation accuracy [56]. If measured dose is extrapolated from patients with individual dosimetry to similar patients, the additional extrapolation error has to be taken into account as well.

To enable time efficient contouring in large epidemiological studies, a useful further development of the atlas would be its implementation in machine-learning based autosegmentation software based on a larger data set of gold standard contours.

## Conclusions

Studying contouring agreement for a heart atlas specifically developed for retrospective cardiac dosimetry in epidemiologic cohort studies, we showed that geometric surrogate volumes for cardiac structures yield better agreement than anatomical structure definitions, notably for the anterior myocardium versus anterior descending arteries. Very small substructures like the valves show poor agreement, limiting the possibility of developing specific dose–response functions for associated late effects.

## Supplementary Information

**Additional file 1.** Heart atlas. Detailed illustrated contouring instructions for all defined heart structures. Sample CT slice with contrast enhancement and visible anatomical structures as well as heart atlas structures.

**Additional file 2.** Supplementary methods and data. Detailed instructions how measures of interobserver variation were calculated. Additional figures with contouring variations shown on CT slices. Additional figures showing individual data for all structures, patients, and observers on structure volume and dosimetric parameters.

## Data Availability

The datasets during and/or analysed during the current study available from the corresponding author on reasonable request.

## Abbreviations

ASD: Average surface distance
CV: Coefficient of variation
DCOM: Distance between centers of mass
DSC: Dice similarity coefficient
HD: Hausdorff distance
ICC: Intraclass correlation coefficient
JSC: Jaccard similarity coefficient
