# Supplementary material for: Heart atlas for retrospective cardiac dosimetry: a multi-institutional study on interobserver contouring variations and their dosimetric impact
Source: Radiat Oncol. 2021 Dec 20;16:241. doi: 10.1186/s13014-021-01965-5 (PMC8691015; doi:10.1186/s13014-021-01965-5)
Supplement: Supplementary file 1 — Additional file 1. Heart atlas. Detailed illustrated contouring instructions for all defined heart structures. Sample CT slice with contrast enhancement and visible anatomical structures as well as heart atlas structures. [file 13014_2021_1965_MOESM1_ESM.pdf]

Heart atlas for retrospective cardiac dosimetry: A multi-institutional study on interobserver contouring variations and their dosimetric impact

Februrary 2021

Supplementary Material A
(HEART ATLAS)

Content

This contouring atlas is designed for retrospective cardiac dosimetry. Aside from the complete heart, it provides six surrogate volumes for functional cardiac structures. The atlas is not designed for individual treatment planning.

The main purpose of this atlas is to improve inter observer contouring agreement in studies using low-resolution CT images without contrast enhancement. Hence, non-anatomical approximations based on simple geometric volumes are used.

Structures

**HEART**	Complete Heart. This corresponds to the definition of the heart by the RTOG Breast Cancer Atlas for Radiation Therapy Planning.
**AOV**	Geometric surrogate volume for the aortic valve.
**PULV**	Geometric surrogate volume for the pulmonary valve.
**MY**	Surrogate volume for the heart wall. It aims to include the pericardium, coronary arteries and the myocardium without the ventricular septum.
**AMYL**	Left anterior sub volume of MY. It aims to contain the left main coronary artery and the left anterior descending artery.
**AMYR**	Right anterior sub volume of MY.<br>It aims to contain the right coronary artery and the sinuatrial node.
**DEEP**	Deep cardiac volume. It would represent non-superficial cardiac structures, that cannot be visualized in low-resolution CT such as AV-node, interventricular septum or mitral valve.

HEART

(complete heart)

CT window level: -200 HU - +300 HU. The visible transition from mediastinum to lung tissue strongly depends on the lower limit of the CT window.

Contouring HEART ***(orange)*** in the given CT window is mandatory ***(a, b, c)***.

Most cranial CT slice ***(a)*** containing HEART: Slice defined by the greatest diameter of the right pulmonary artery ***(a: 1)***.

Most caudal CT slice containing HEART: Last slice with a clear separation between mediastinal tissue and liver tissue.

Concave contours should be avoided.

The pericardium will be contoured once it is visible ***(c: 2)***.

The tissue connecting the mediastinum to the sternum will be excluded ***(a, b: 3)***.

Trachea, bronchi, esophagus, descending aorta, hila and pulmonary veins will be excluded if distinguishable.

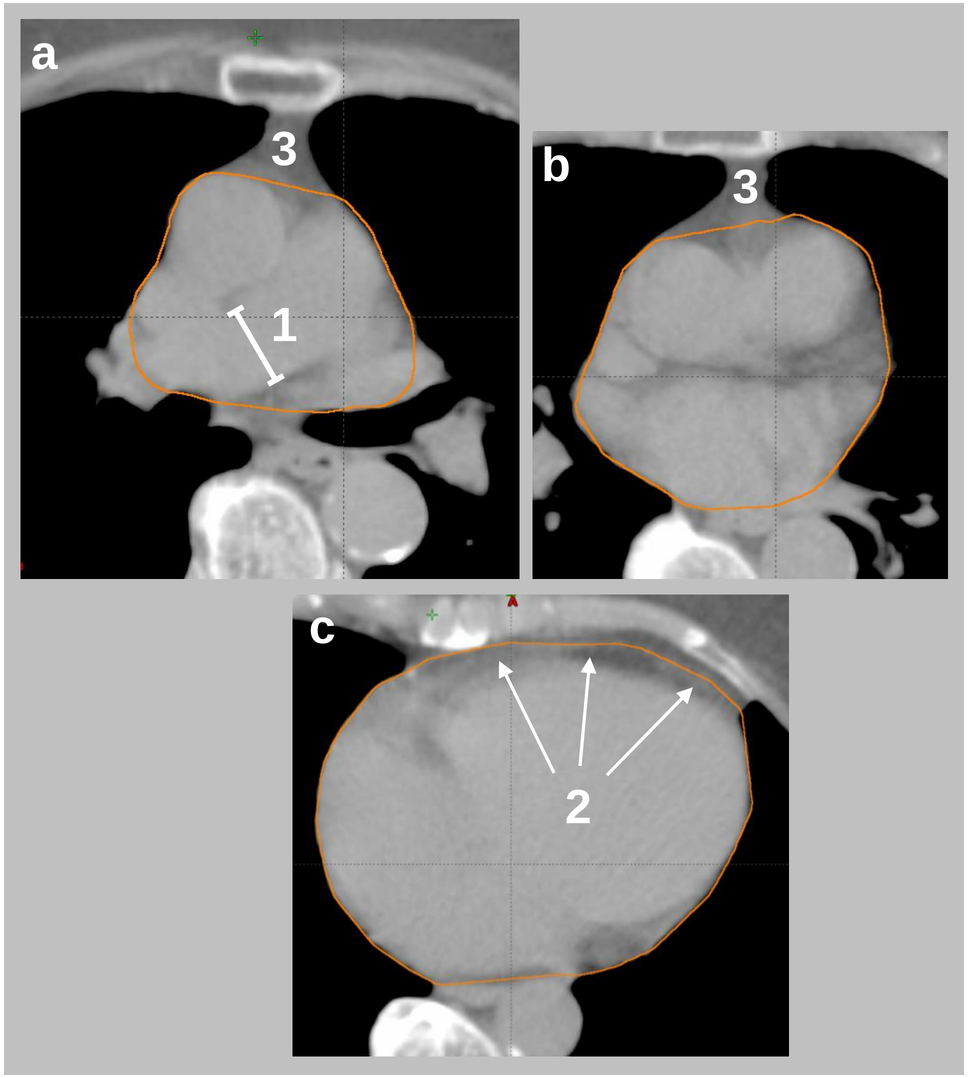

AOV

(aortic valve surrogate)

First, it is necessary to choose a CT slice ***(a, b)***

- where the left main stem *or*
- the proximal segment of the left anterior descending artery *or*
- the right coronary artery is visible.

Should there be no proximal coronary arteries visible, a slice below the right pulmonary artery with the greatest visible gap between pulmonary trunk ***(1)*** and left atrium ***(2)*** will be chosen.

The aortic root will be contoured in the chosen slice ***(a, b: red)***.

That contour will be copied ***(d)*** to a CT slice 3 cm caudal ***(e: white)***. There, only the 6 o´clock segment will be retained ***(e: red)***.

Then, the volume AOV will be interpolated.

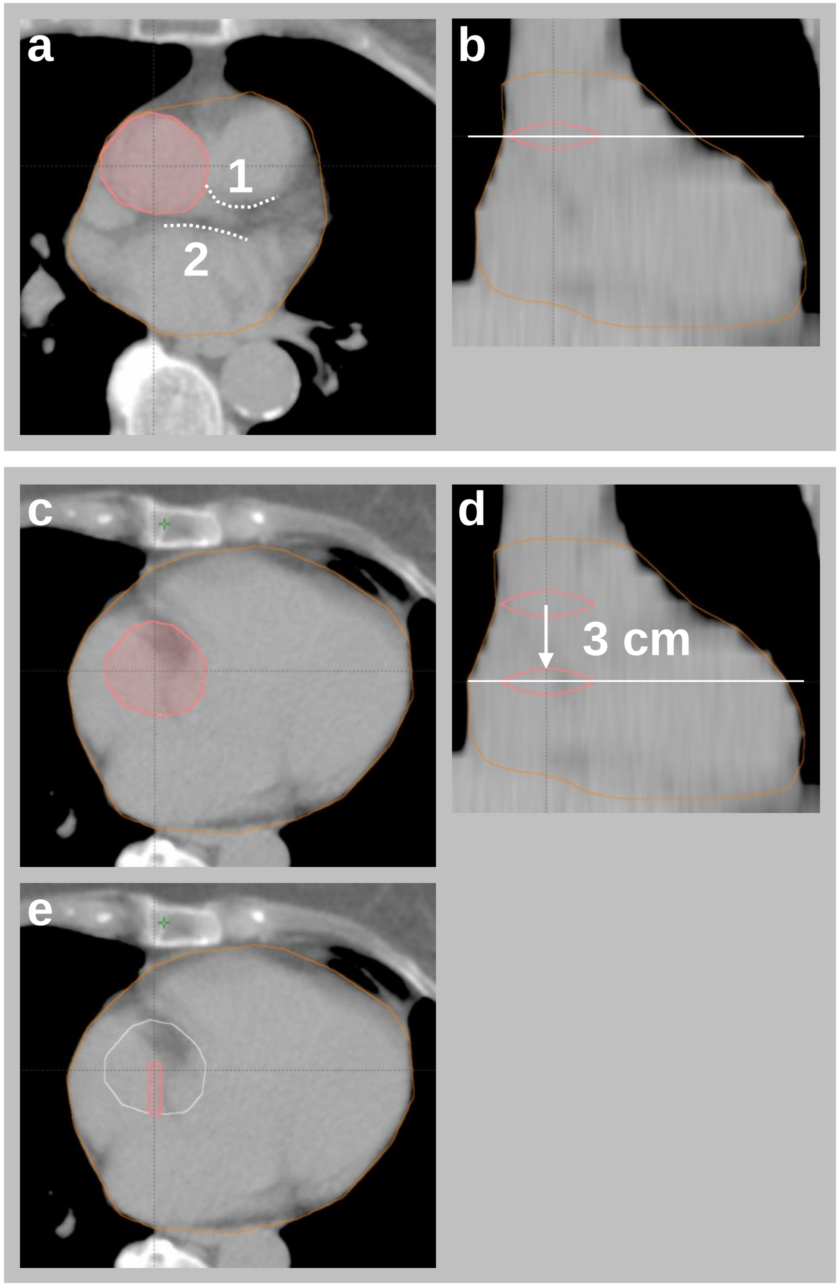

PULV

(pulmonary valve surrogate)

CT slice used in the definition of volume AOV where the aortic root ***(a, b: red)*** was contoured is chosen again.

A point ***(a, b: point)*** will be found that is equidistant to the contours of HEART and AOV ***(a: arrows)***.

The point might be eccentric to the pulmonary trunk, but not outside of it.

The structure PULV ***(b: light blue)*** will be spherically expanded until it nearly reaches the HEART or the AOV contour (keep approximately 2 mm distance).

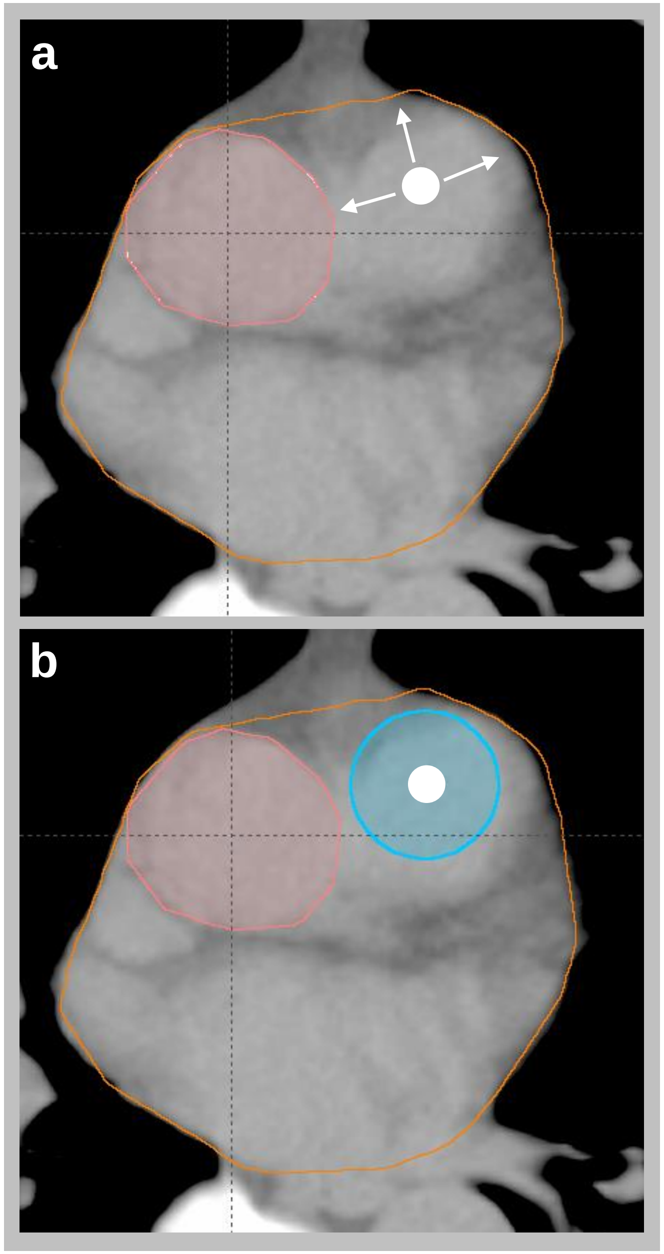

MY

(myocardium surrogate)

At first, the structure MY will be generated as a copy of HEART.

Then, all CT slices above the structure AOV will be deleted.

At last, a wall structure with a thickness of 1cm will be extracted.

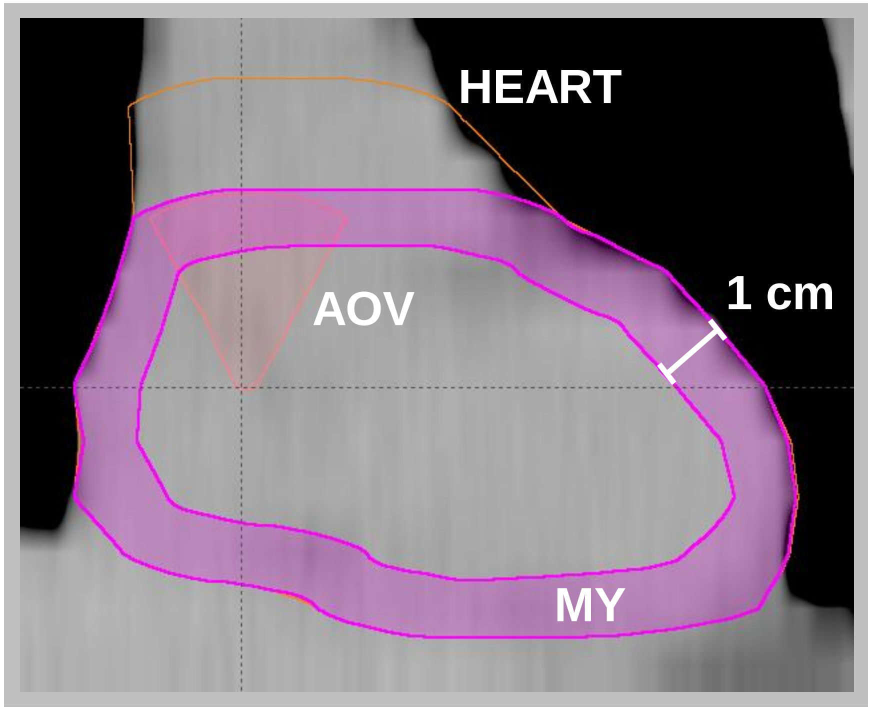

AMYL

(left anterior myoycardium surrogate)

Again, the CT slice used for defining volume AOV with the aortic root will be chosen ***(a, b)***.

A rectangular help structure *(HELP_L, white)* is contoured. Its right border ***(a: arrowhead)*** is tangent to the aortic root ***(a: red)***. The collinear extension of its dorsal border is tangent to the dorsal border of the aortic root ***(a: arrowhead)***.

Now, a slice 2 cm cranial to the most caudal slice of HEART is chosen ***(c, d)***.

HELP_L will be copied here ***(c: white)***. The dorsal border will be adjusted to intersect with MY perpendicularly ***(e: white dotted line)***.

HELP_L will be interpolated.

AMYL is generated by the overlap of MY and HELP_L.

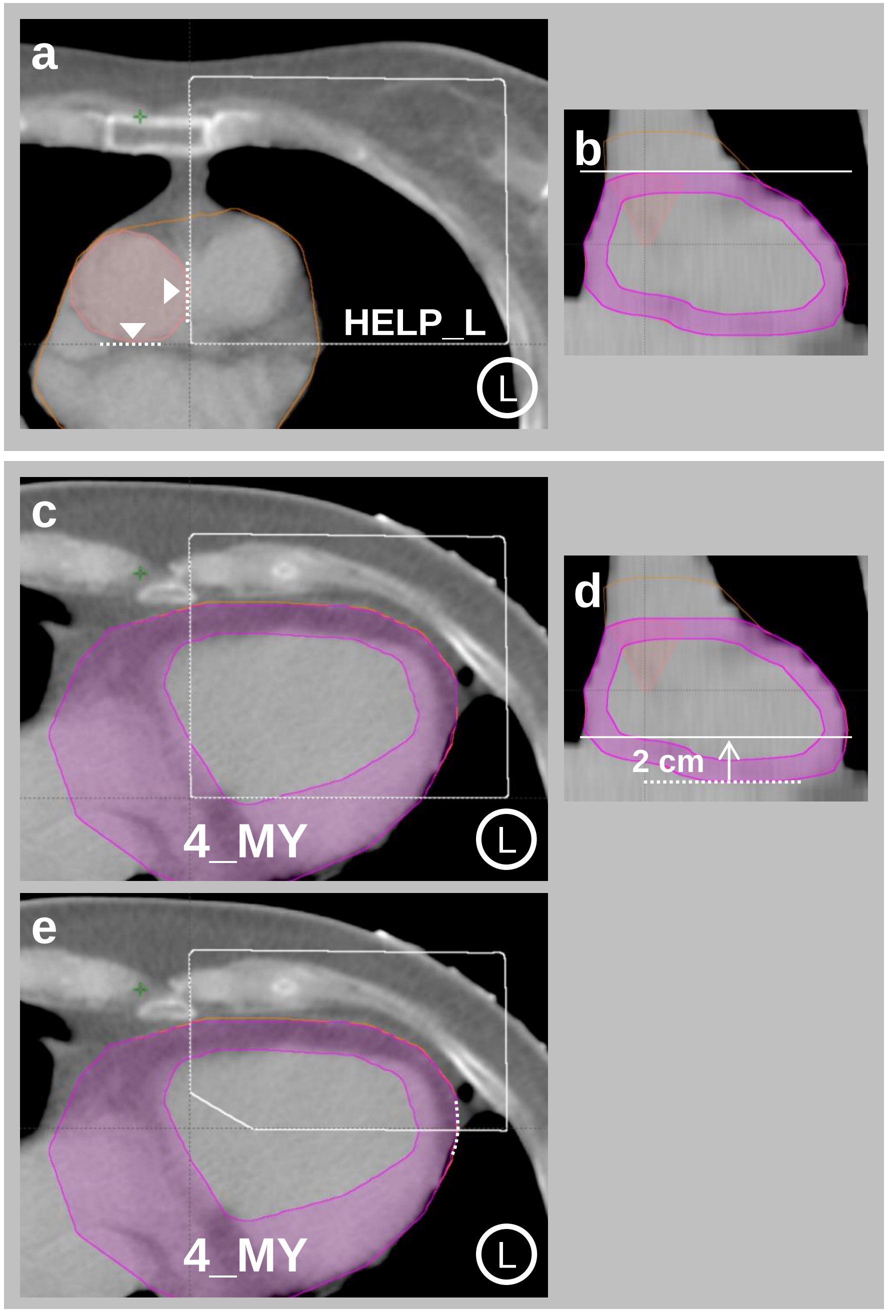

AMYR

(right anterior myocardium surrogate)

The CT slice used for defining volume AOV with the aortic root is chosen ***(a, b)***.

A rectangular contour is generated (HELP_R). Its left border is tangent to the left border of the aortic root ***(a: arrowhead)***. Its dorsal border is tangent to the dorsal border of the aortic root ***(a: arrowhead)***.

Now, a slice 2 cm cranial to the most caudal slice of HEART is chosen ***(c, d)***.

HELP_R will be copied here ***(c: white)***. The dorsal border will be adjusted to intersect with MY perpendicularly ***(e: white dotted line)***.

HELP_R will be interpolated.

AMYL is generated by the overlap of MY and HELP_R.

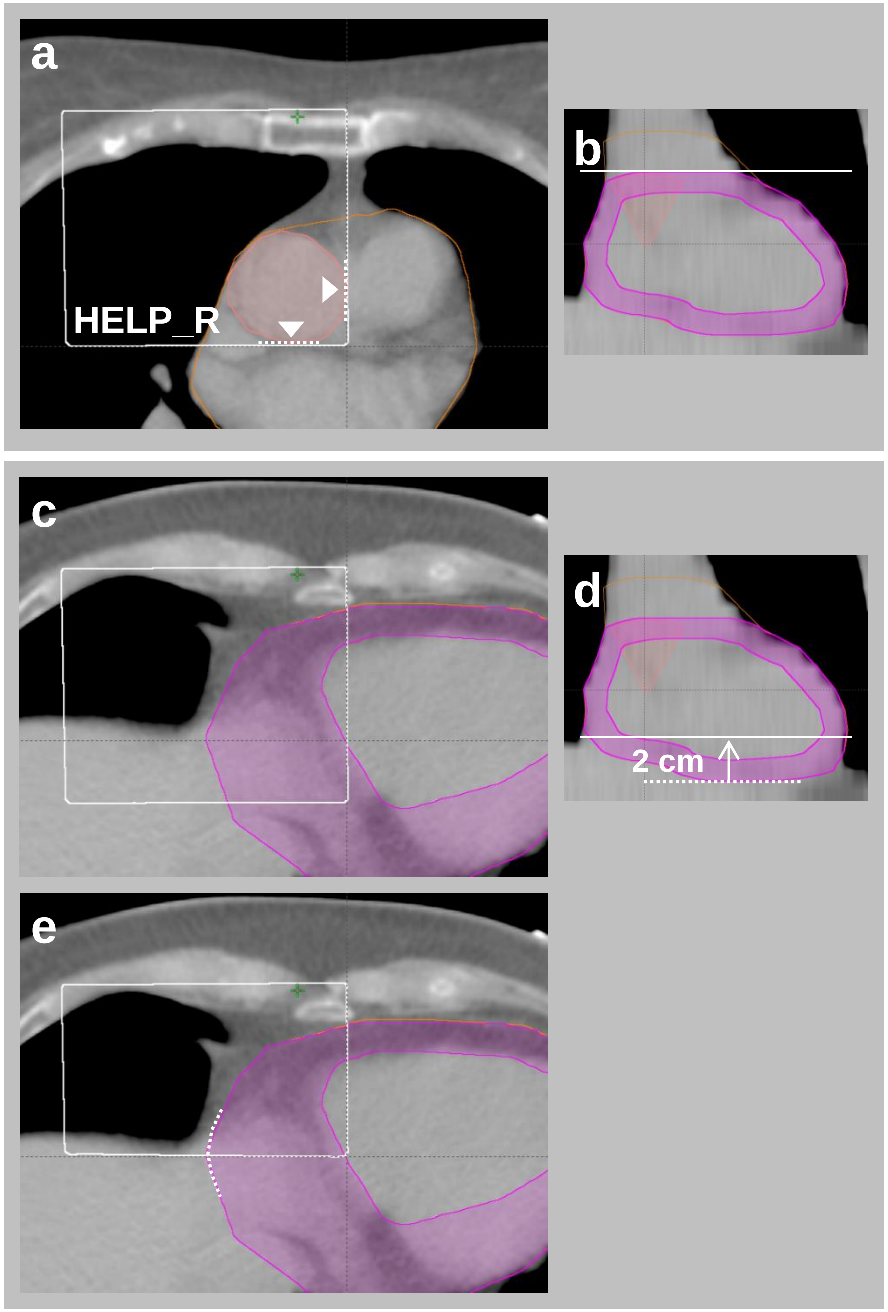

DEEP

To generate a robust surrogate for deep cardiac structures, the structure HEART will be shrinked by 2cm in all directions

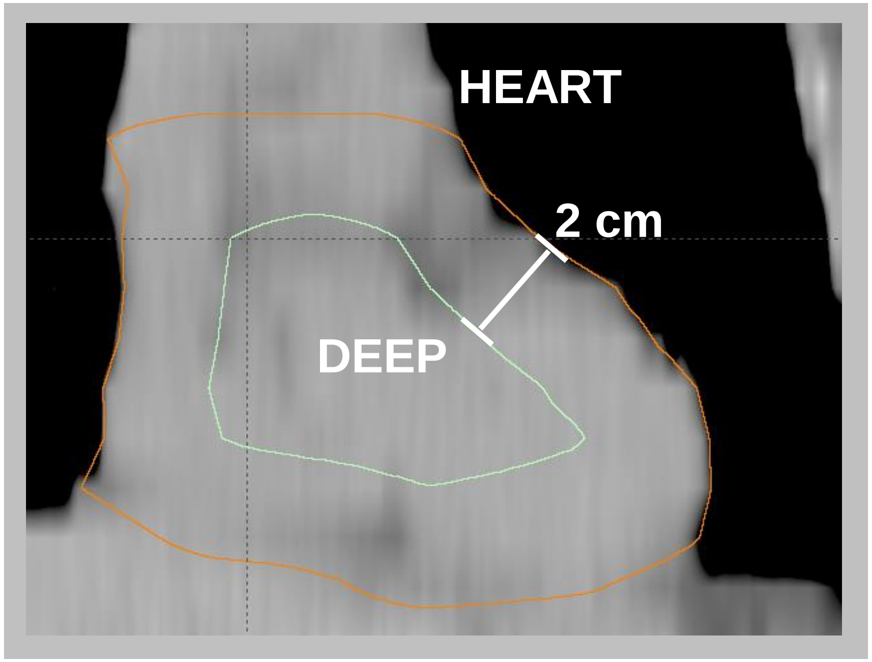

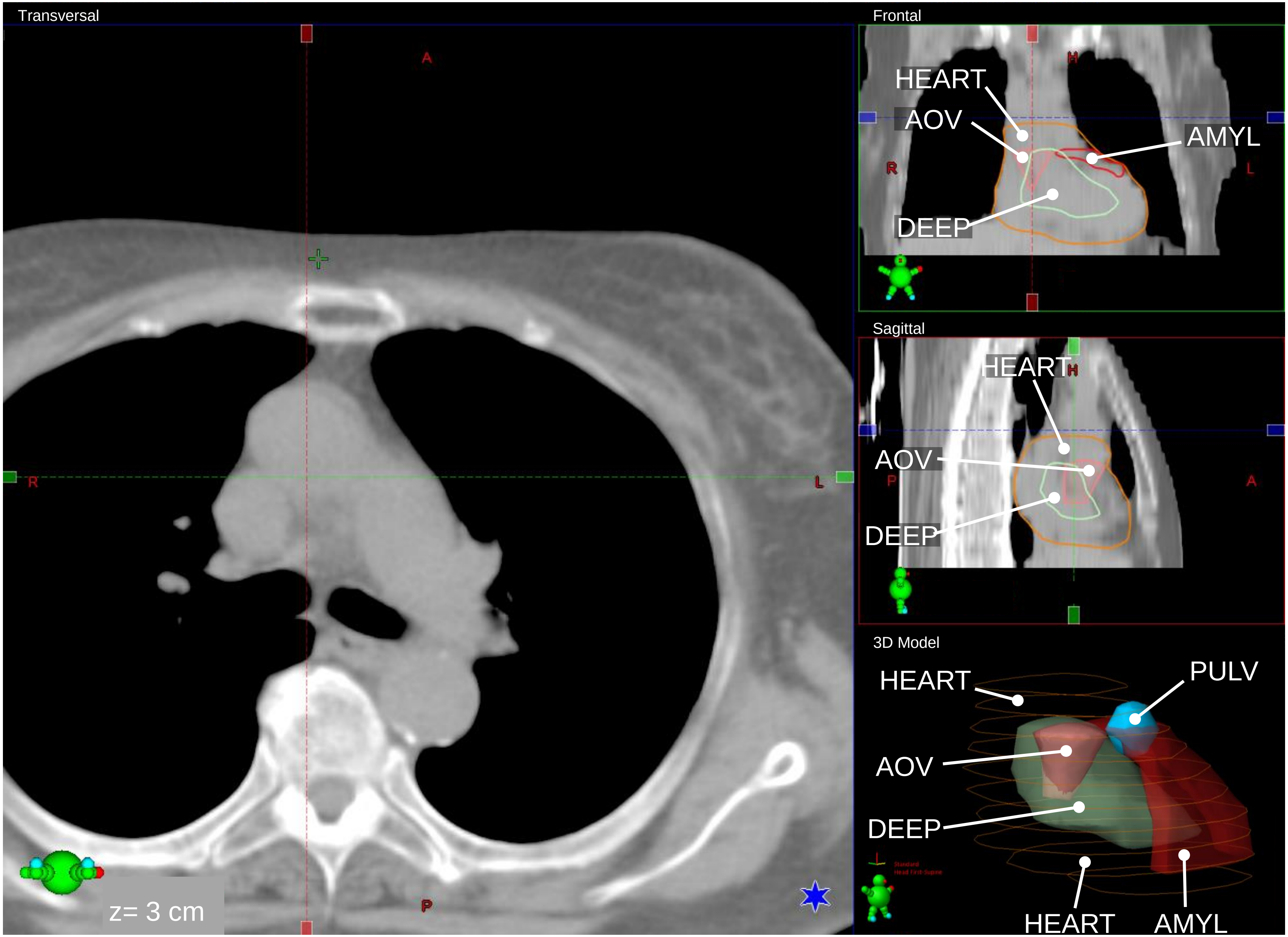
Transversal
A
R
L
P
z= 3 cm
Frontal
HEART
AOV
AMYL
DEEP
H
R
L
Sagittal
HEART
H
AOV
P
A
DEEP
3D Model
HEART
PULV
AOV
DEEP
HEART
AMYL

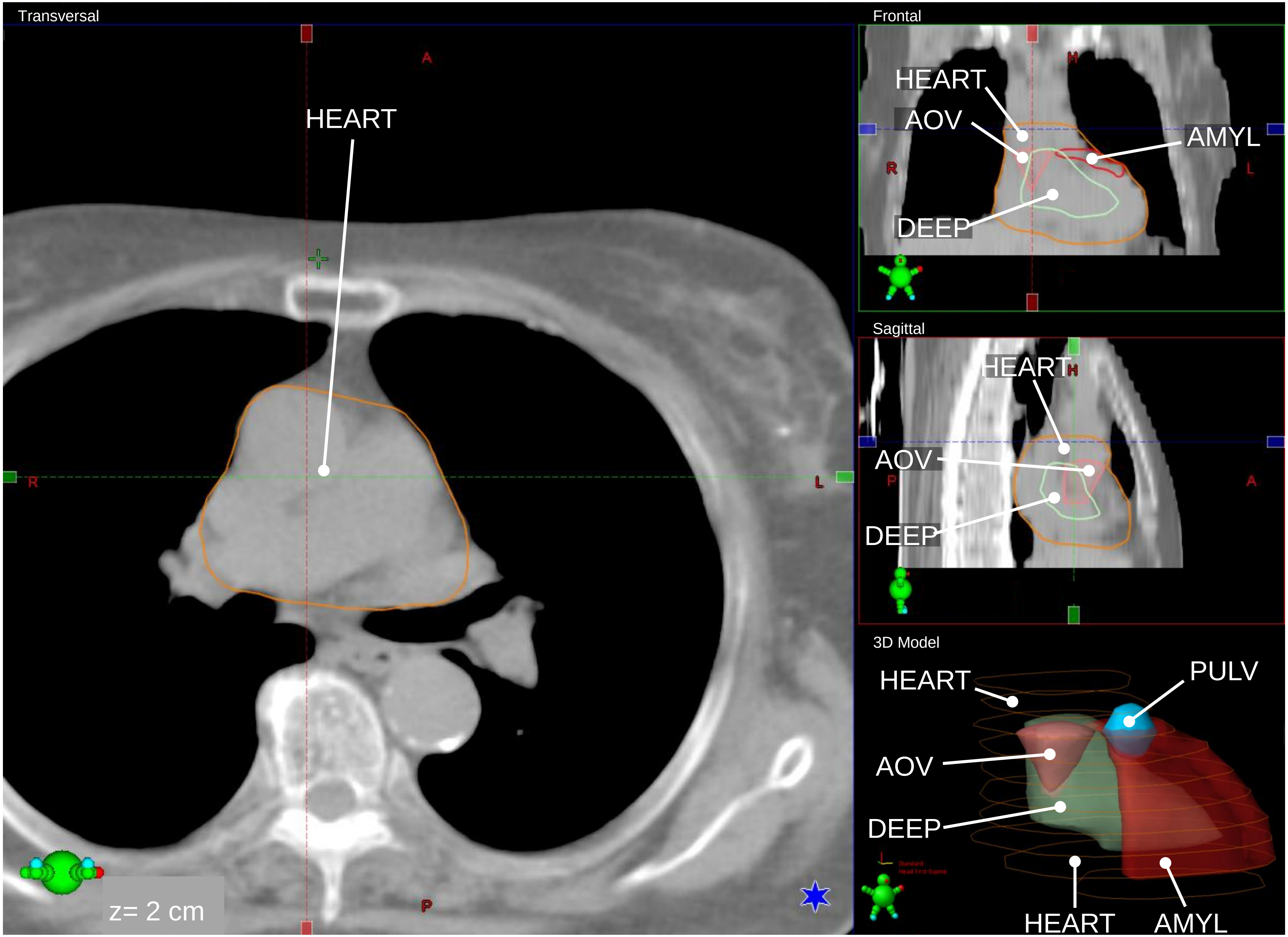
Transversal
A
HEART
R
L
P
z= 2 cm
Frontal
H
HEART
AOV
AMYL
R
L
DEEP
Sagittal
HEART
H
AOV
P
A
DEEP
3D Model
HEART
PULV
AOV
DEEP
HEART
AMYL

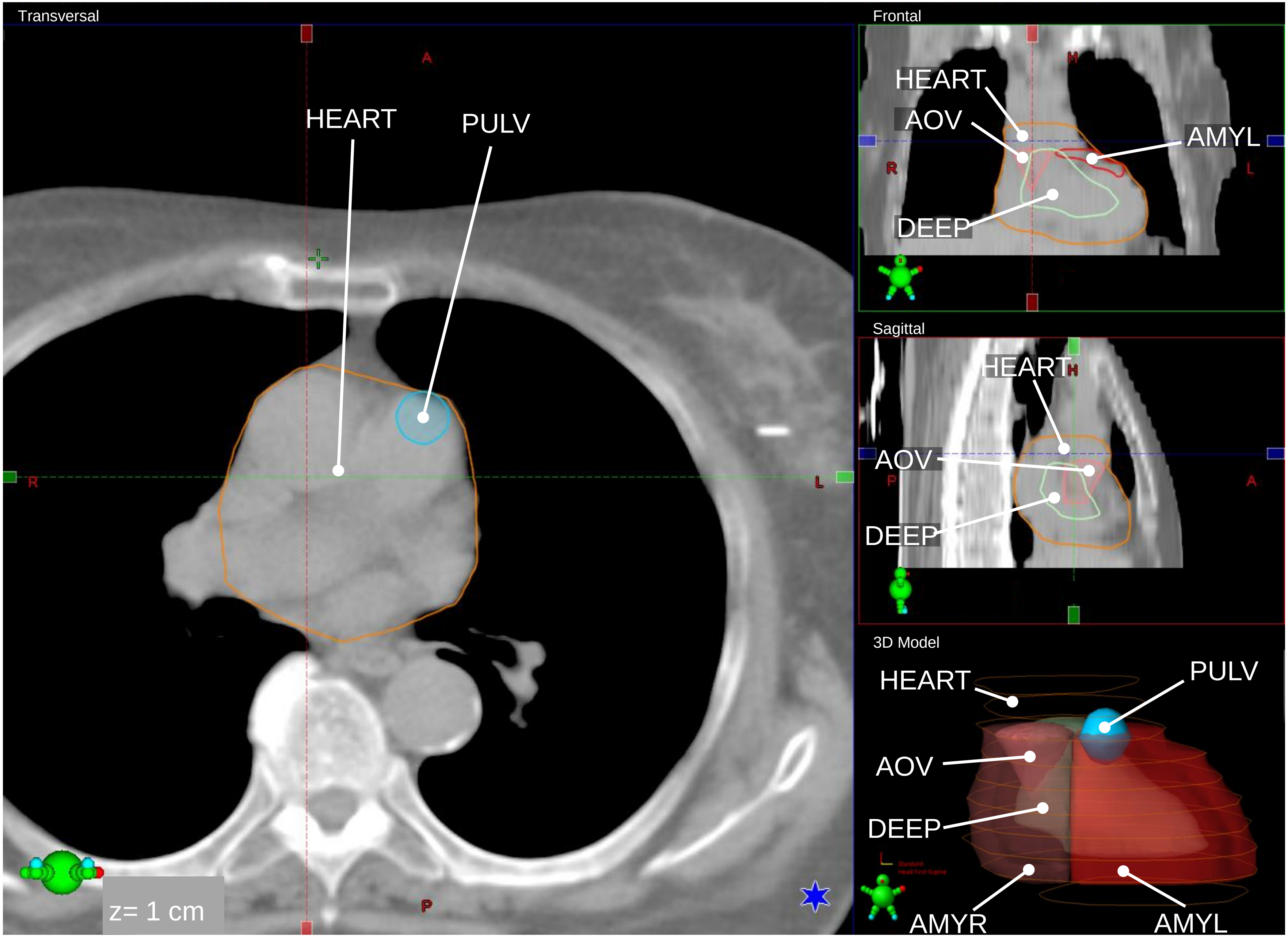

Transversal
A
HEART
PULV
R
L
P
z= 1 cm
Frontal
HEART
AOV
AMYL
H
R
L
DEEP
Sagittal
HEART
H
AOV
P
A
DEEP
3D Model
HEART
PULV
AOV
DEEP
AMYR
AMYL

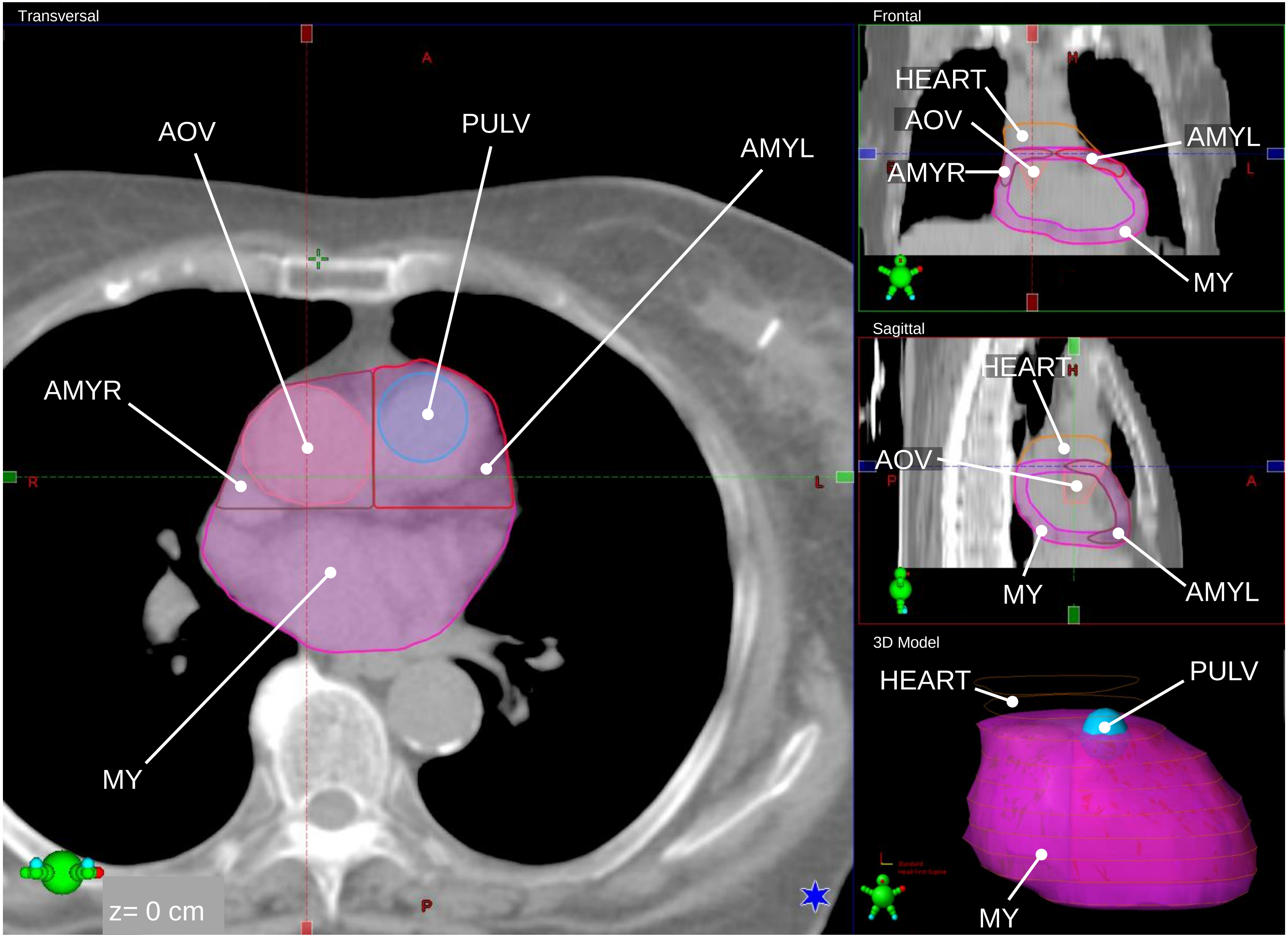
Transversal
A
AOV
PULV
AMYL
AMYR
R
L
MY
P
z= 0 cm
Frontal
HEART
AOV
AMYL
AMYR
H
L
MY
Sagittal
HEART
H
AOV
P
A
MY
AMYL
3D Model
HEART
PULV
MY

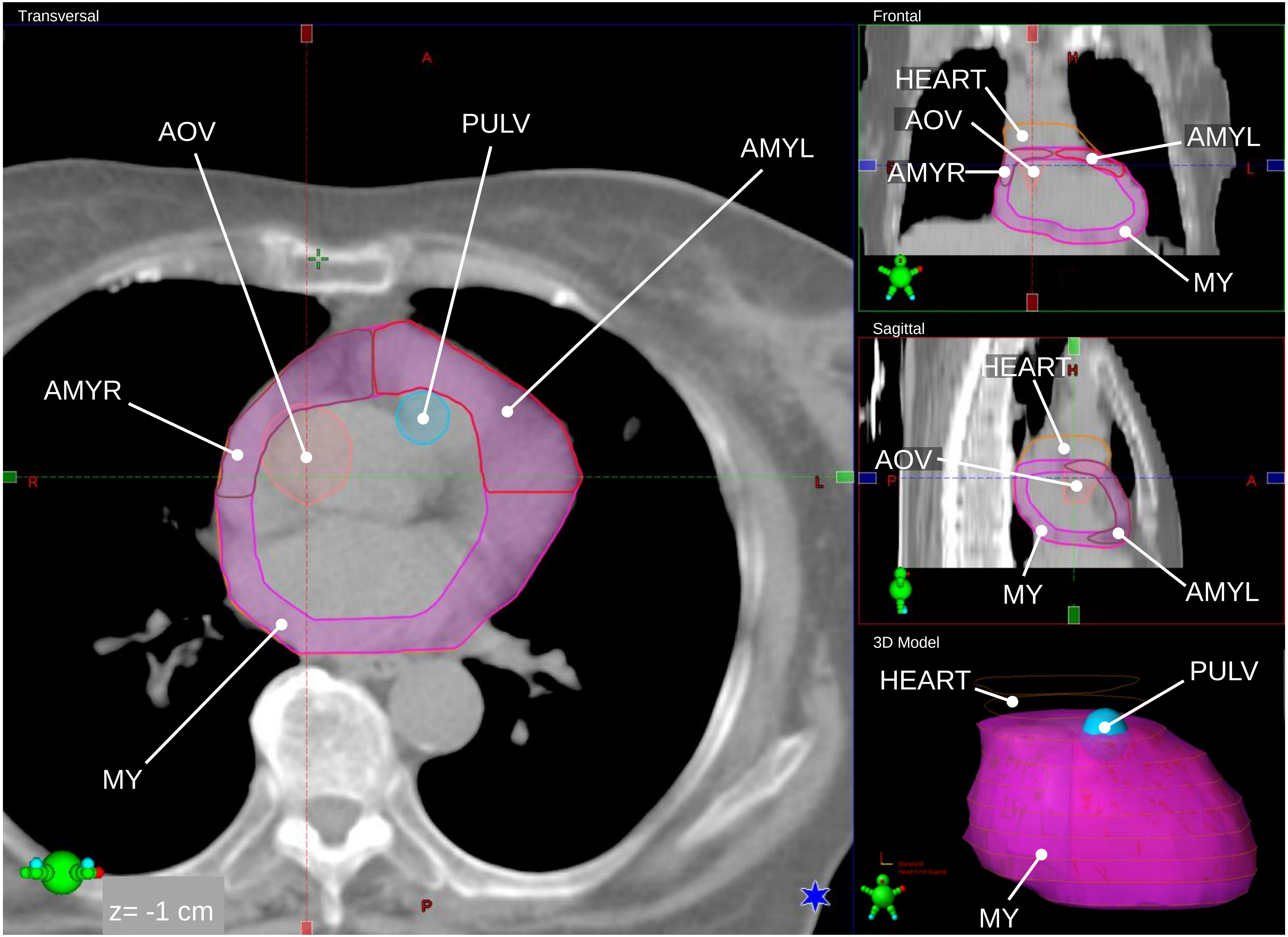
Transversal
A
AOV
PULV
AMYL
AMYR
R
L
MY
P
z= -1 cm
Frontal
HEART
AOV
AMYL
AMYR
MY
Sagittal
HEART
AOV
MY
AMYL
3D Model
HEART
PULV
MY

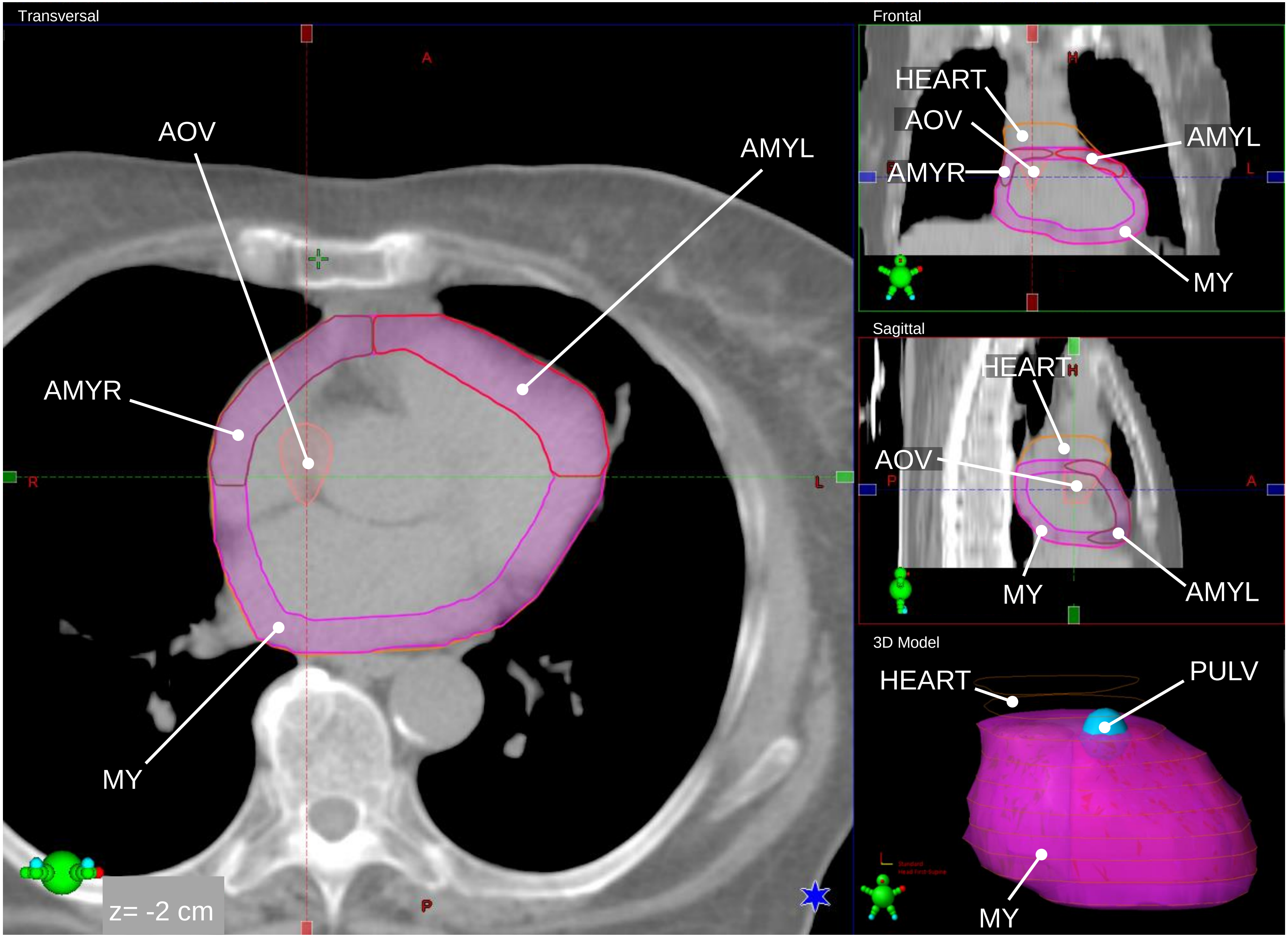
Transversal
AOV
AMYL
AMYR
MY
z= -2 cm
A
R
L
P
Frontal
HEART
AOV
AMYL
AMYR
MY
Sagittal
HEART
AOV
MY
AMYL
3D Model
HEART
PULV
MY

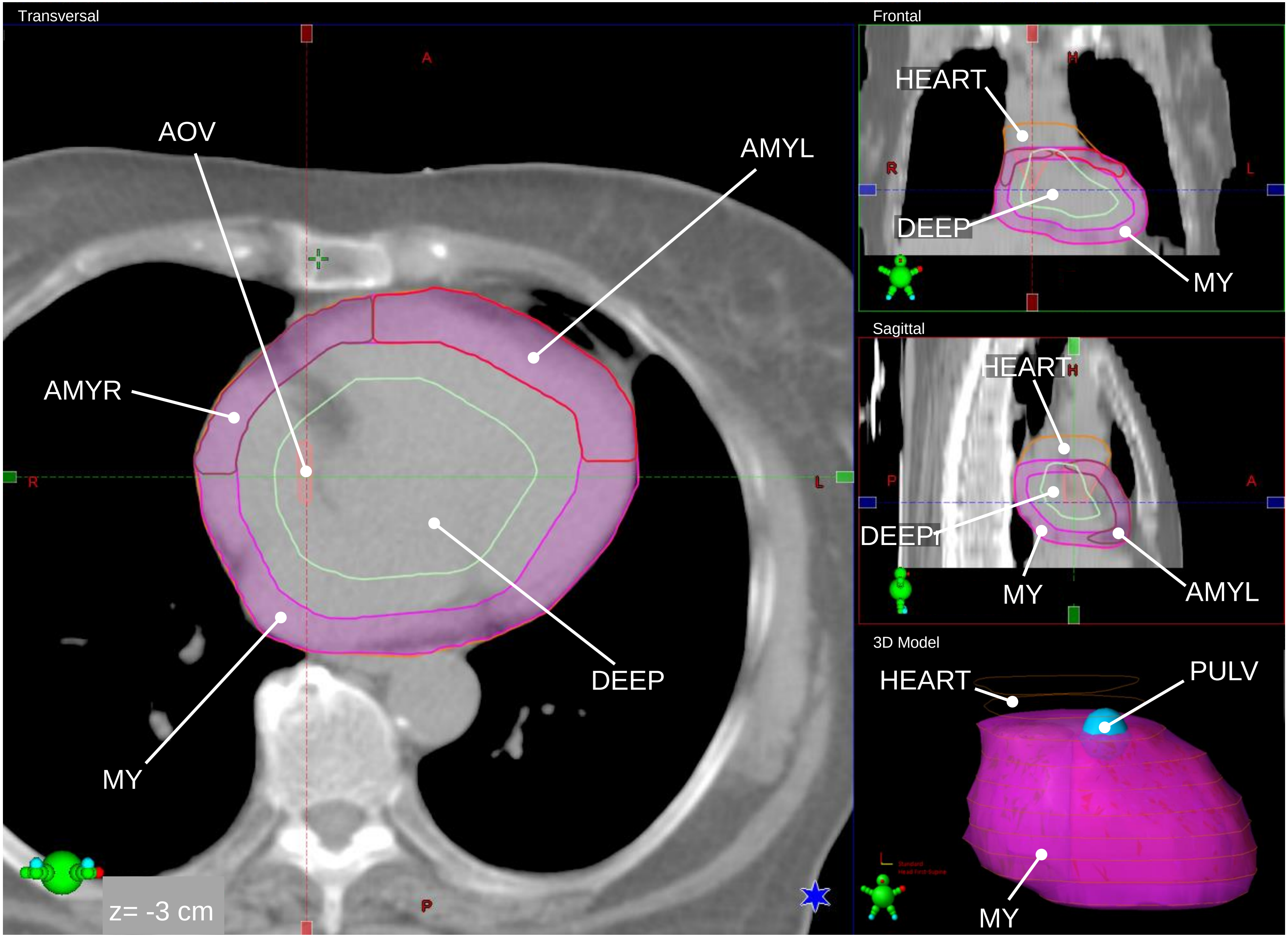
Transversal
AOV
AMYL
AMYR
DEEP
MY
A
R
L
P
z= -3 cm
Frontal
HEART
H
R
L
DEEP
MY
Sagittal
HEART
H
P
A
DEEP
MY
AMYL
3D Model
HEART
PULV
MY

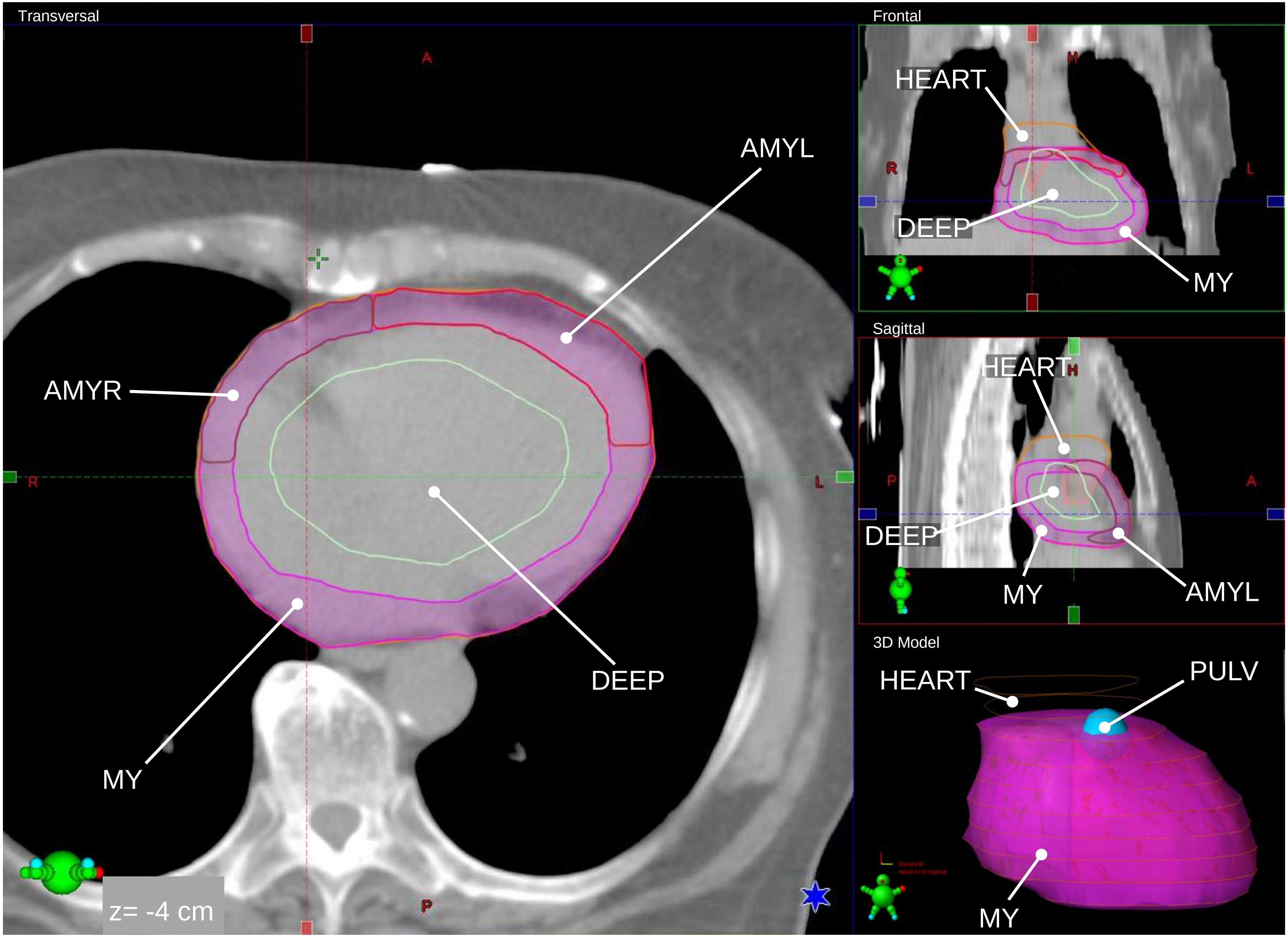
Transversal
Frontal
Sagittal
3D Model
AMYL
AMYR
DEEP
MY
HEART
PULV
A
P
R
L
H
z= -4 cm

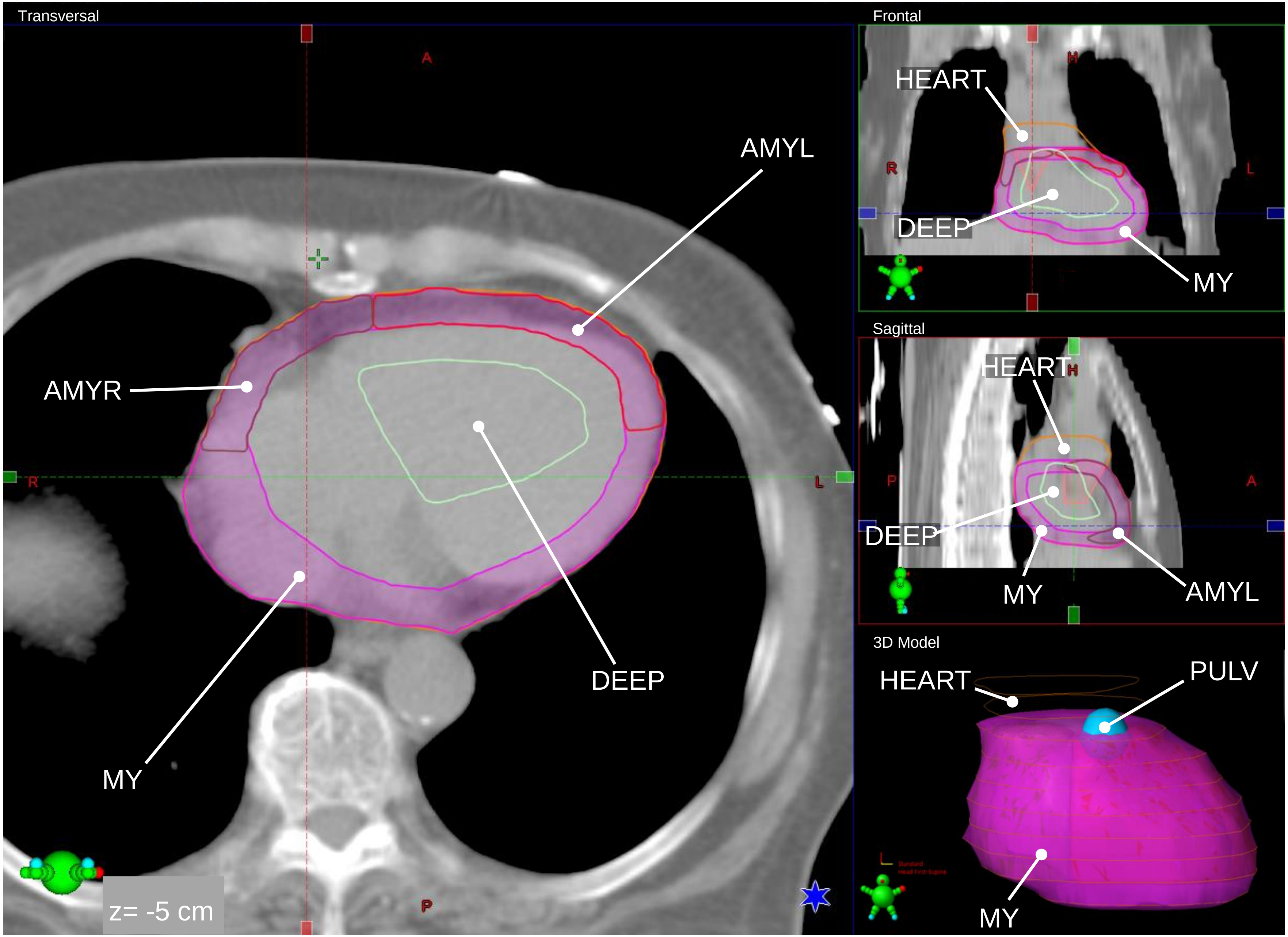
Transversal
AMYL
AMYR
DEEP
MY
A
R
L
P
z= -5 cm
Frontal
HEART
DEEP
MY
H
R
L
Sagittal
HEART
DEEP
MY
AMYL
H
P
A
3D Model
HEART
PULV
MY

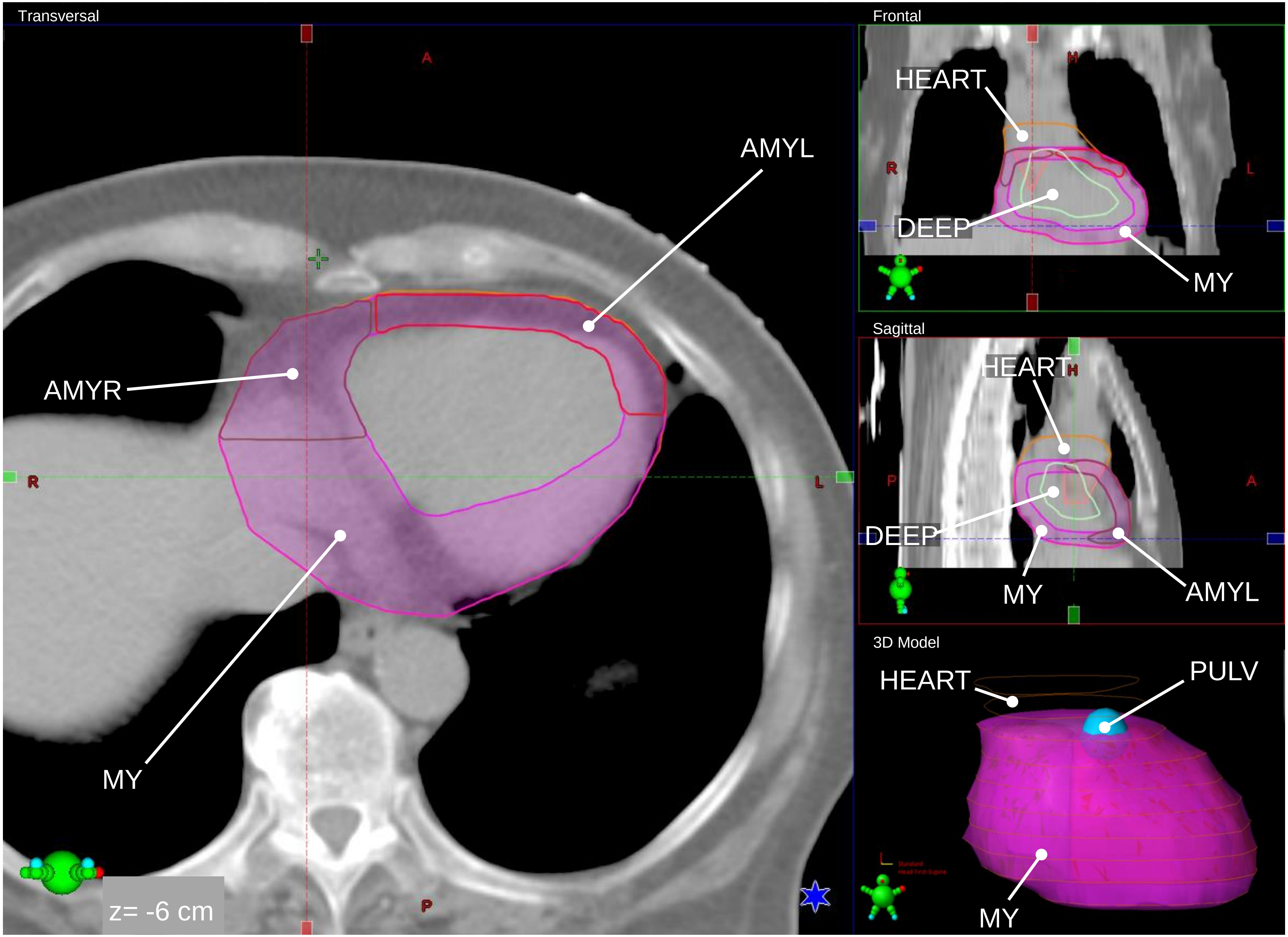
Transversal
A
AMYL
AMYR
R
L
MY
P
z= -6 cm
Frontal
HEART
H
R
L
DEEP
MY
Sagittal
HEART
H
P
A
DEEP
MY
AMYL
3D Model
HEART
PULV
MY

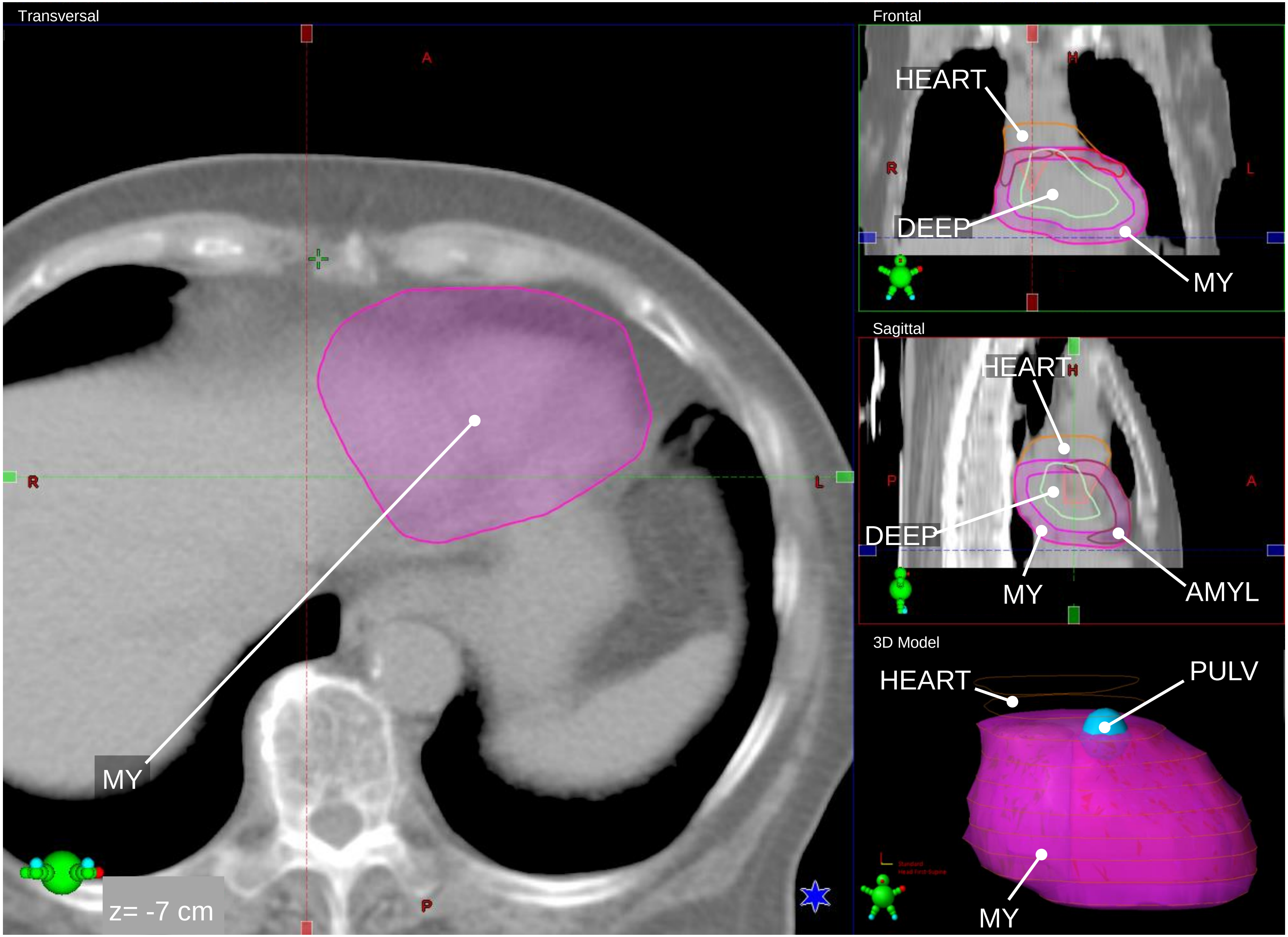
Transversal
A
R
L
P
MY
z= -7 cm
Frontal
HEART
H
R
L
DEEP
MY
Sagittal
HEART
H
P
A
DEEP
MY
AMYL
3D Model
HEART
PULV
MY

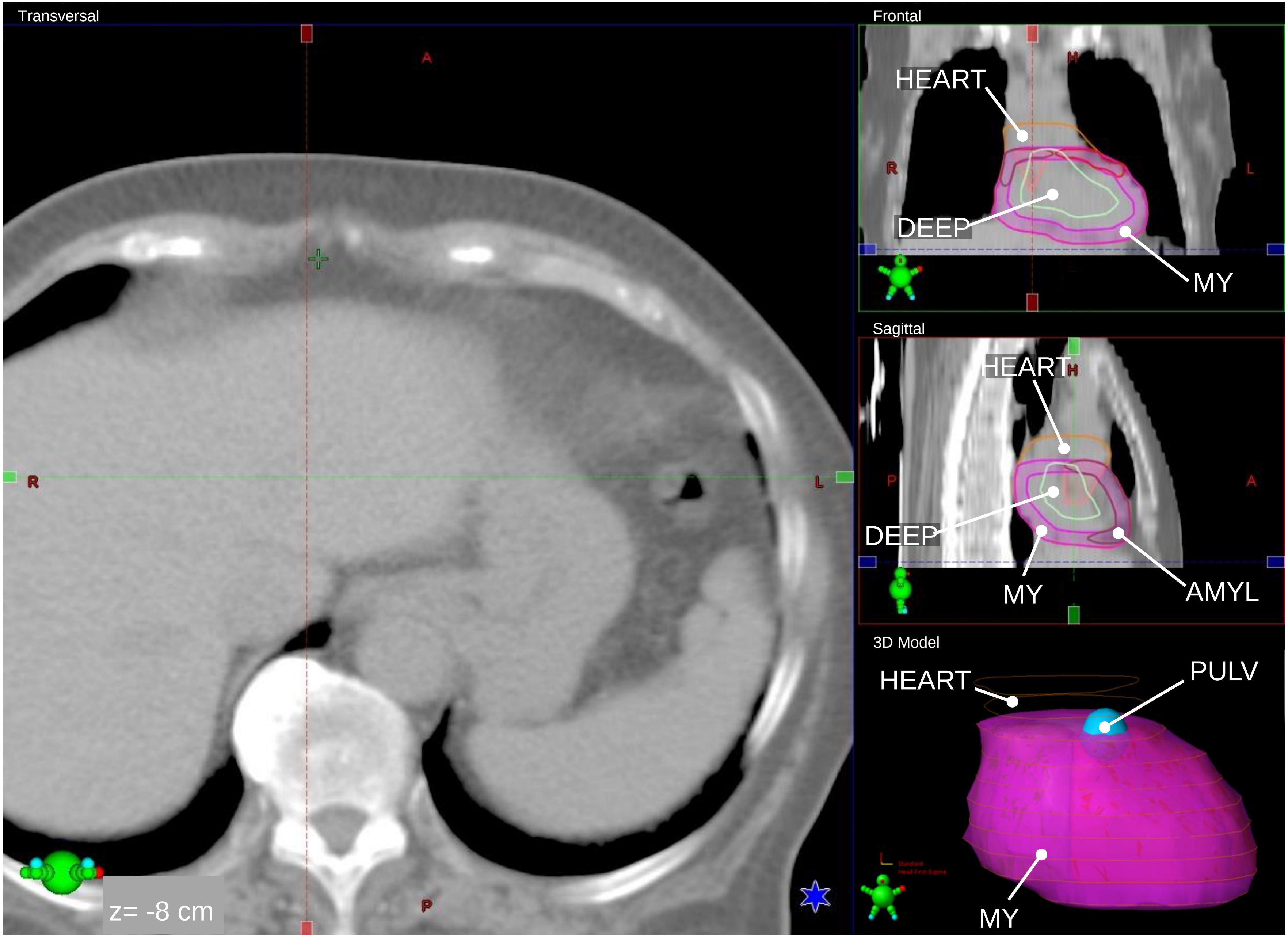
Transversal
A
R
L
P
z= -8 cm
Frontal
HEART
DEEP
MY
H
R
L
Sagittal
HEART
H
P
A
DEEP
MY
AMYL
3D Model
HEART
PULV
MY

Heart atlas structures in a diagnostic CT scan of the heart with contrast enhancement: HEART (orange), MY (violet) , AMYL (red), AMYR (brown), DEEP (green).

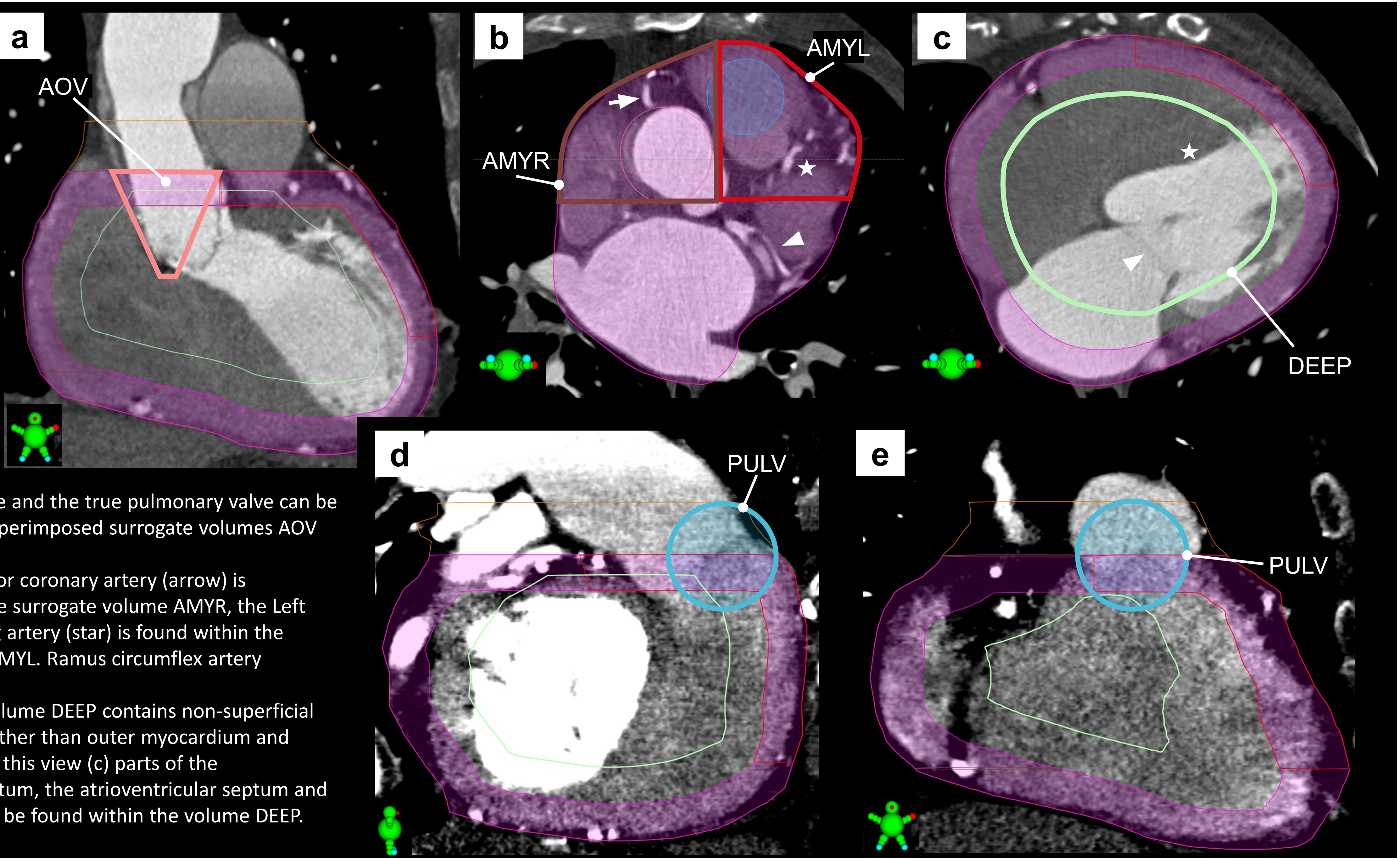

The true aortic valve and the true pulmonary valve can be compared to the superimposed surrogate volumes AOV (a) and PULV (d, e).
(b) The Right anterior coronary artery (arrow) is encompassed by the surrogate volume AMYR, the Left anterior descending artery (star) is found within the surrogate volume AMYL. Ramus circumflex artery (arrowhead).
(c) The surrogate volume DEEP contains non-superficial cardiac structures other than outer myocardium and coronary vessels. In this view (c) parts of the interventricular septum, the atrioventricular septum and the mitral valve can be found within the volume DEEP.
