# Supplementary material for: Heart atlas for retrospective cardiac dosimetry: a multi-institutional study on interobserver contouring variations and their dosimetric impact
Source: Radiat Oncol. 2021 Dec 20;16:241. doi: 10.1186/s13014-021-01965-5 (PMC8691015; doi:10.1186/s13014-021-01965-5)
Supplement: Supplementary file 2 — Additional file 2. Supplementary methods and data. Detailed instructions how measures of interobserver variation were calculated. Additional figures with contouring variations shown on CT slices. Additional figures showing individual data for all structures, patients, and observers on structure volume and dosimetric parameters. [file 13014_2021_1965_MOESM2_ESM.pdf]

Heart atlas for retrospective cardiac dosimetry: A multi-institutional study on interobserver contouring variations and their dosimetric impact

Marcus Stockinger[1], Heiko Karle[1], Hannes Rennau[2], Sabine Sebb[2], Ulrich Wolf[3], Julia Remmele[3], Sandra Bührdel[4], Detlef Bartkowiak[4], Maria Blettner[5], Heinz Schmidberger[1], and Daniel Wollschläger[5]

[1]Department of Radiation Oncology, University Medical Center Mainz, Mainz, Germany
[2]Department of Radiation Oncology, University Hospital Rostock, Rostock, Germany
[3]Department of Radiation Oncology, University Hospital Leipzig, Leipzig, Germany
[4]Department of Radiation Oncology, University Hospital Ulm, Ulm, Germany
[5]Institute of Medical Biostatistics, Epidemiology and Informatics, University Medical Center Mainz, Mainz, Germany; wollschlaeger@uni-mainz.de

February 2021

Supplementary Material

1 Supplementary methods

We here provide details on calculating spatial and dosimetric agreement measures (Babalola et al., 2009; Fotina, Lütgendorf-Caucig, Stock, Pötter, & Georg, 2012; Hanna, Hounsell, & O'Sullivan, 2010).

1.1 Spatial agreement measures

Each observer's delineation of each heart atlas structure for each patient was exported from Varian Eclipse (version 15, Varian Medical Systems, Palo Alto, CA) as a three-dimensional surface triangle mesh using the Eclipse Scripting API. The mesh resolution was 1 mm. In addition, a cumulative dose-volume histogram (DVH) was exported for each of the delineated structures with dose bins of 10 cGy.

In the following description, we refer to patients $i = 1, \ldots, N$ (with $N = 16$); heart atlas structures $j = 1, \ldots, J$ (with $J = 7$); observers $k = 1, \ldots, K$ (with $K = 6$), and observer pairs $l = 1, \ldots, L$ (with $L = \binom{K}{2} = 15$).

Since no observer had been designated as gold standard, the spatial agreement between 2 delineations of the same structure was calculated using several measures for each of the 15 observer pairs, and then averaged over observer pairs.

1.1.1 Pairwise distance-based measures

Pairwise distance-based agreement measures were calculated using the libigl software (Jacobson & Panozzo, 2021) based on the exported surface triangle meshes.

Distance between centers of mass (DCOM) For a possibly non-convex mesh A, the center of mass (COM) vector $\overline{\boldsymbol{a}} = (\overline{x}_a, \overline{y}_a, \overline{z}_a)^\top$ was calculated using a surface integral (Nürnberg, 2013). For two meshes A and B with respective COM $\overline{\boldsymbol{a}}$ and $\overline{\boldsymbol{b}}$, DCOM is defined as the Euclidean distance $\|\cdot\|_2$ between $\overline{\boldsymbol{a}}$ and $\overline{\boldsymbol{b}}$:

$$\text{DCOM} = \|\overline{\boldsymbol{a}} - \overline{\boldsymbol{b}}\|_2 = \sqrt{(\overline{x}_a - \overline{x}_b)^2 + (\overline{y}_a - \overline{y}_b)^2 + (\overline{z}_a - \overline{z}_b)^2}$$

The average pairwise $\overline{\text{DCOM}}_j$ for one heart atlas structure j was calculated by averaging DCOM_{ijl} over observer pairs l and patients i:

$$\overline{\text{DCOM}}_j = \frac{1}{N} \sum_{i=1}^{N} \frac{1}{L} \sum_{l=1}^{L} \text{DCOM}_{ijl}$$

Average surface distance (ASD) Given mesh A with vertices $\boldsymbol{a}_r = (x_{a_r}, y_{a_r}, z_{a_r})^\top$ ($r = 1, \dots, R$) and mesh B with vertices $\boldsymbol{b}_s = (x_{b_s}, y_{b_s}, z_{b_s})^\top$ ($s = 1, \dots, S$), the Euclidean distance between one pair of vertices – one from each mesh – is:

$$d(\boldsymbol{a}_r, \boldsymbol{b}_s) = \|\boldsymbol{a}_r - \boldsymbol{b}_s\|_2 = \sqrt{(x_{a_r} - x_{b_s})^2 + (y_{a_r} - y_{b_s})^2 + (z_{a_r} - z_{b_s})^2}$$

For a single vertex $\boldsymbol{a}_r$ from A, the Euclidean distance to mesh B is defined as the distance from $\boldsymbol{a}_r$ to the closest vertex of B:

$$d(\boldsymbol{a}_r, B) = \min(d(\boldsymbol{a}_r, \boldsymbol{b}_s)) \quad , s = 1, \dots, S$$

ASD between meshes A and B is defined as the average of all the distances of one vertex to the respective other mesh:

$$\text{ASD} = \frac{\sum_{r=1}^{R} d(\boldsymbol{a}_r, B) + \sum_{s=1}^{S} d(\boldsymbol{b}_s, A)}{R + S}$$

Hausdorff distance (HD) For meshes A and B, HD is defined as the average of the longest distances from A to B, and from B to A:

$$\text{HD} = \frac{\max(d(\boldsymbol{a}_r, B)) + \max(d(\boldsymbol{b}_s, A))}{2} \quad , r = 1, \dots, R; s = 1, \dots, S$$

The average pairwise $\overline{\text{ASD}}_j$ and $\overline{\text{HD}}_j$ for one heart atlas structure j were calculated by averaging ASD_{ijl} and HD_{ijl}, respectively, over observer pairs l and patients i.

1.1.2 Pairwise volume-overlap measures

To calculate volume-overlap measures for each observer-pair, we used the libigl software (Jacobson & Panozzo, 2021) to perform Boolean operations on the exported surface triangle meshes, and to calculate mesh volumes via the divergence theorem (Nürnberg, 2013).

Jaccard similarity coefficient (JSC) For structures A and B, their intersection $A \cap B$ has volume $|A \cap B|$ and their union $A \cup B$ has volume $|A \cup B|$. JSC (Jaccard, 1912) is then defined as:

$$\text{JSC} = \frac{|A \cap B|}{|A \cup B|}$$

Dice similarity coefficient (DSC) DSC (Dice, 1945) for structures A and B with respective volumes $|A|$ and $|B|$ is defined as:

$$\text{DSC} = \frac{2 \cdot |A \cap B|}{|A| + |B|}$$

The average pairwise $\overline{\text{JSC}}_j$ and $\overline{\text{DSC}}_j$ for one heart atlas structure j were calculated by averaging JSC_{ijl} and DSC_{ijl}, respectively, over observer pairs l and patients i.

1.1.3 Agreement for structure volume

The DVHmetrics package (Wollschlaeger & Karle, 2020) for the statistical environment R (R Core Team, 2021) was used to calculate overall agreement measures for the volume of each delineated structure based on the cumulative DVHs exported from Varian Eclipse.

Coefficient of variation (CV) A log-normal distribution was assumed for structure volume. The CV, defined as the ratio of the standard deviation to the mean, is then given by $\sqrt{e^{\sigma^2} - 1}$, with σ^2 being the variance of the $\log_e$-transformed volume values.

The structure-specific CV_j based on data for all patients were derived from fitting a Bayesian log-normal regression model for the observed volume values using package brms (Bürkner, 2017) for the statistical environment R. The covariate was a factor indexing all possible combinations of patient and structure, and the model allowed for structure-specific error variances σ_j^2. Based on the estimated $\hat{\sigma}_j^2$, the structure-specific CV_j were calculated as $\text{CV}_j = 100 \cdot \sqrt{e^{\hat{\sigma}j^2} - 1}$. The 95% credible intervals for CV_j were derived from the posterior distribution of the $\hat{\sigma}_j$.

As a sensitivity analysis, we descriptively calculated the structure-specific $\overline{\text{CV}}_j$ as the ratio of the square root of the mean of the variances s_{ij}^2 of the volume values in structure j and patient i to the average of the volume means $\overline{V}_{ij}$:

$$\overline{\text{CV}}_i = 100 \cdot \frac{\sqrt{\frac{1}{N} \sum_{i=1}^{N} s_{ij}^2}}{\frac{1}{N} \sum_{i=1}^{N} \overline{V}_{ij}}$$

Intraclass correlation coefficient (ICC) For a fixed heart atlas structure j, the variation in volume has two systematic variance components: σ_b^2 for the variance due to patients i (*between*) and σ_w^2 for the variance due to observers k (*within*). The population ICC as a measure for observer agreement is then defined as (Shrout & Fleiss, 1979):

$$\text{ICC} = \frac{\sigma_b^2}{\sigma_b^2 + \sigma_w^2}$$

To estimate ICC, we assume an analysis-of-variance (ANOVA) design with covariate factors *patient* and *observer* whose levels both represent random samples from a larger population. Adopting the naming scheme from Shrout and Fleiss (1979), this is a two-way random design to estimate ICC(2) as a measure for absolute agreement (consistency) among observers.

Given a data sample, the variance components are estimated from two different ANOVAs: The first ANOVA is a one-way design with factor *patient* from which MS_{pat} is calculated, the mean effect sum of squares for *patient*. The second ANOVA is a two-way design with factors *patient*

and *observer* from which we calculate $\mathrm{MS_{obs}}$, the mean effect sum of squares for *observer*, and $\mathrm{MS_{err}}$, the mean error sum of squares. ICC(2) can then be estimated as:

$$\widehat{\mathrm{ICC}}(2)_j = \frac{\mathrm{MS_{pat}} - \mathrm{MS_{err}}}{\mathrm{MS_{pat}} + (K-1)\cdot\mathrm{MS_{err}} + K\cdot\frac{\mathrm{MS_{obs}}-\mathrm{MS_{err}}}{N}}$$

For each structure j, we calculated $\widehat{\mathrm{ICC}}(2)_j$ and the corresponding 95% confidence interval using package psych (Revelle, 2020) for the statistical environment R.

1.2 Dose-based agreement measures

Overall agreement measures for dose metrics in each delineated heart atlas structure were calculated based on the cumulative DVHs exported from Varian Eclipse using the DVHmetrics package (Wollschlaeger & Karle, 2020) for the statistical environment R.

1.2.1 Coefficient of variation (CV)

The structure-specific CV_j for dose metrics DMEAN and D2CC were calculated, respectively, as for structure volume (section 1.1.3).

1.2.2 Standard deviation (SD)

For V5GY as a natural proportion, a beta distribution with parameters μ (mean) and ϕ (precision) was assumed. Structure specific SD_j based on data for all patients were derived from fitting a Bayesian beta regression model for the observed values using package brms. The covariate was a factor indexing all possible combinations of patient and structure, and the model allowed for structure-specific precision parameters ϕ_j. Based on the estimated $\hat{\mu}_j$ and $\hat{\phi}_j$, the structure-specific SD_j were then calculated as $\mathrm{SD}_j = \sqrt{\frac{\hat{\mu}_j\cdot(1-\hat{\mu}_j)}{1+\hat{\phi}_j}}$. The 95% credible intervals for ϕ_j were derived from the posterior distribution of the parameter estimates.

As a sensitivity analysis, we descriptively calculated the structure-speficic $\overline{\mathrm{SD}}_j$ for V5GY as the square root of the arithmetic mean of the empirical variances s_{ij}^2 for one heart atlas structure j from one patient i as $\overline{\mathrm{SD}}_j = \sqrt{\frac{1}{N}\sum_{i=1}^{N}\mathrm{s}_{ij}^2}$.

1.2.3 Intraclass correlation coefficient (ICC)

The respective structure-specific ICC_j for dose metrics DMEAN, D2CC, and V5GY were calculated as for structure volume (section 1.1.3).

2 Supplementary data

2.1 Interobserver variations in contour delineation

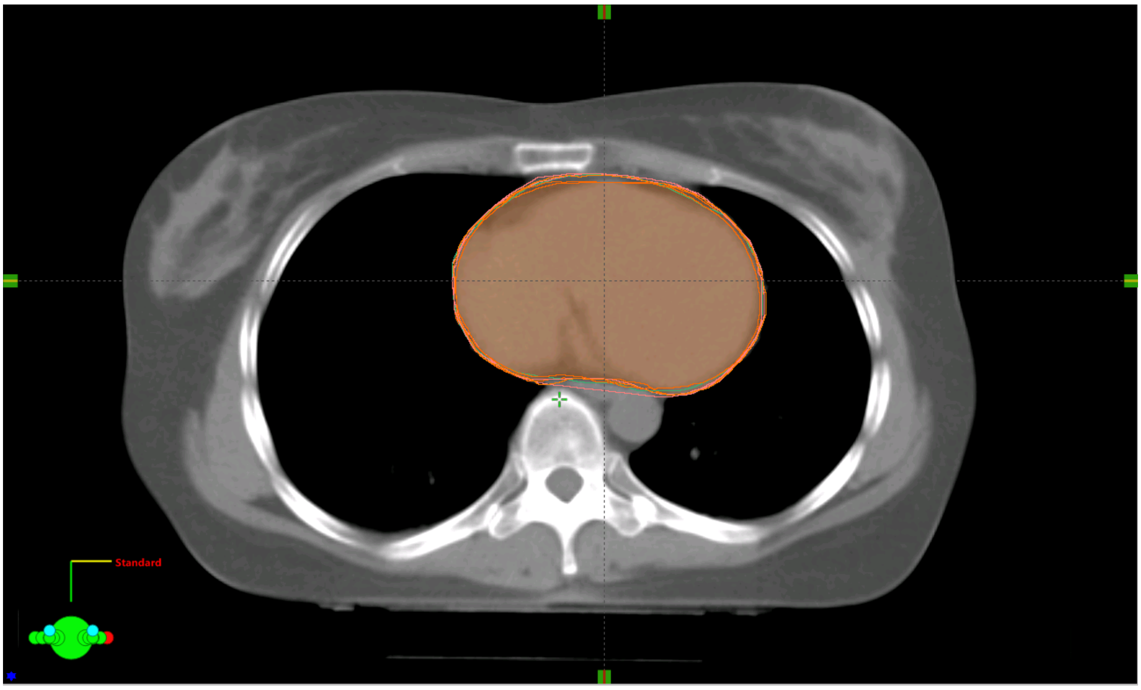

Figure S1: Contour delineations for the complete heart – axial plane.

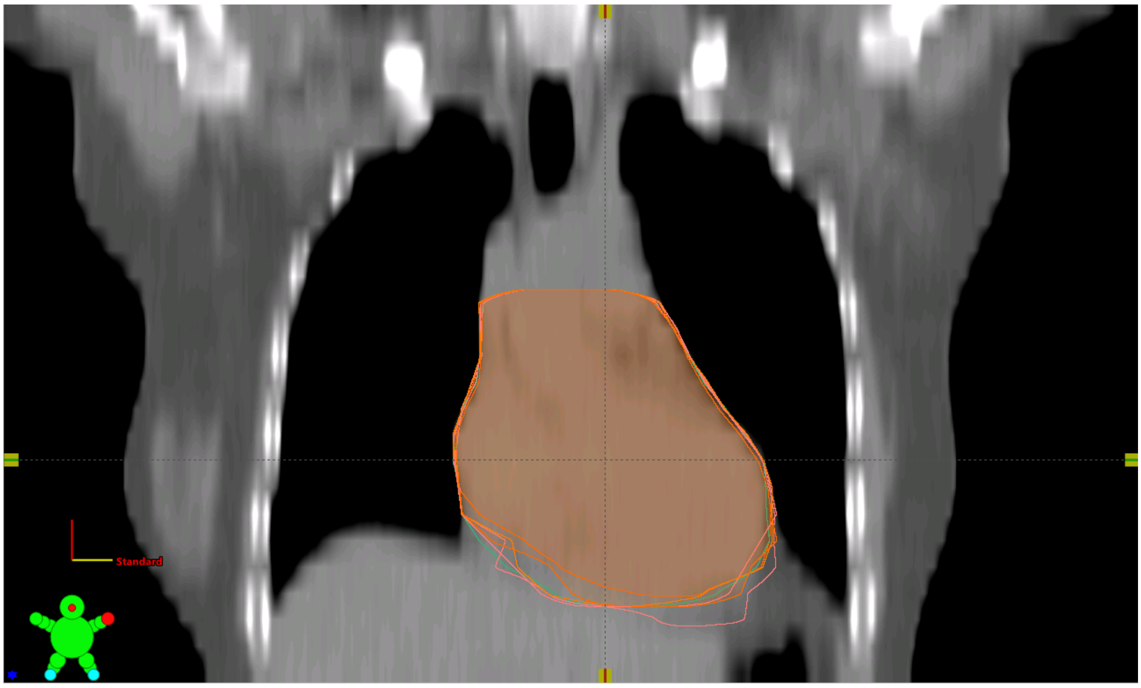

Figure S2: Contour delineations for the complete heart – coronary plane.

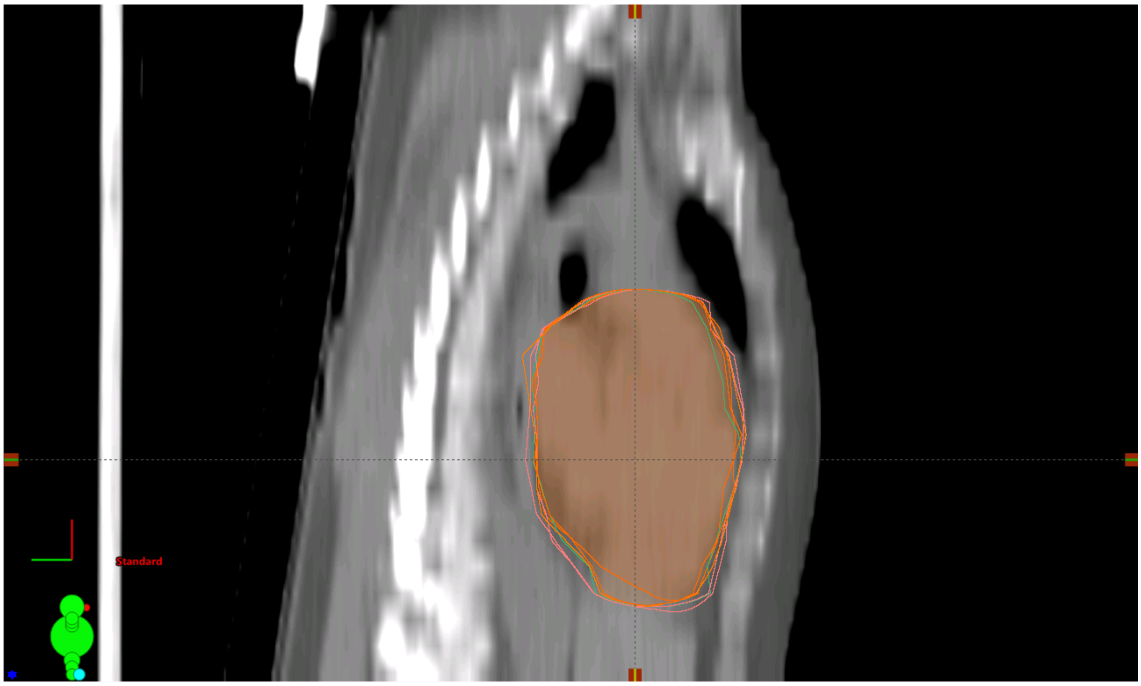

Figure S3: Contour delineations for the complete heart – sagittal plane.

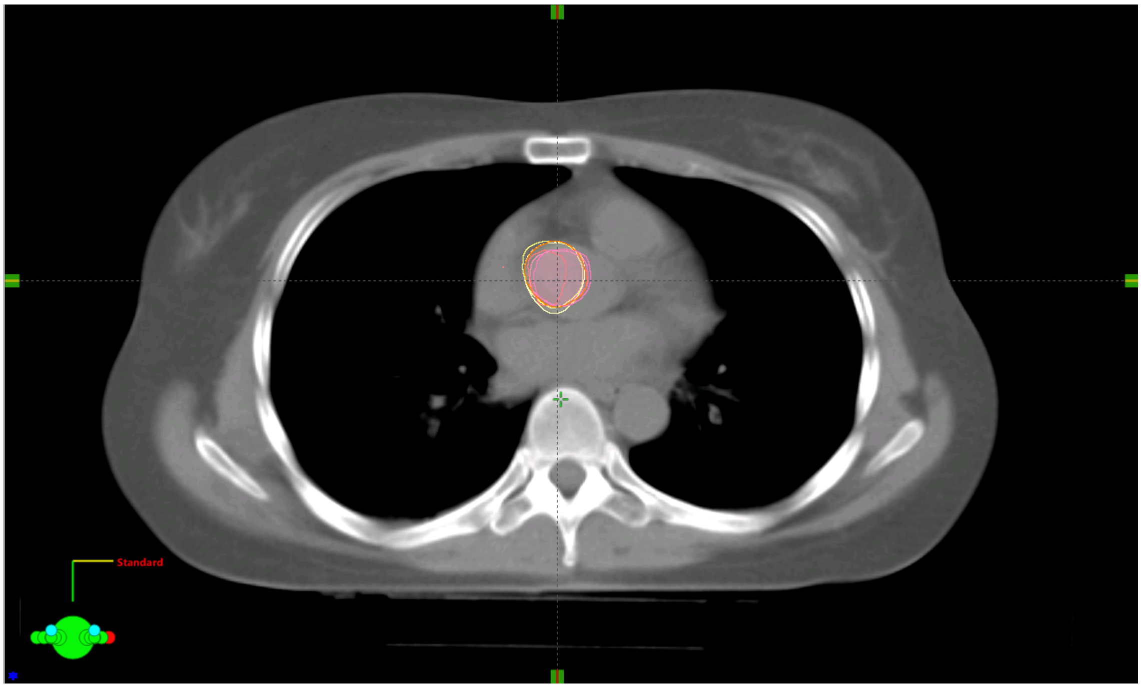

Figure S4: Contour delineations for the aortic valve – axial plane.

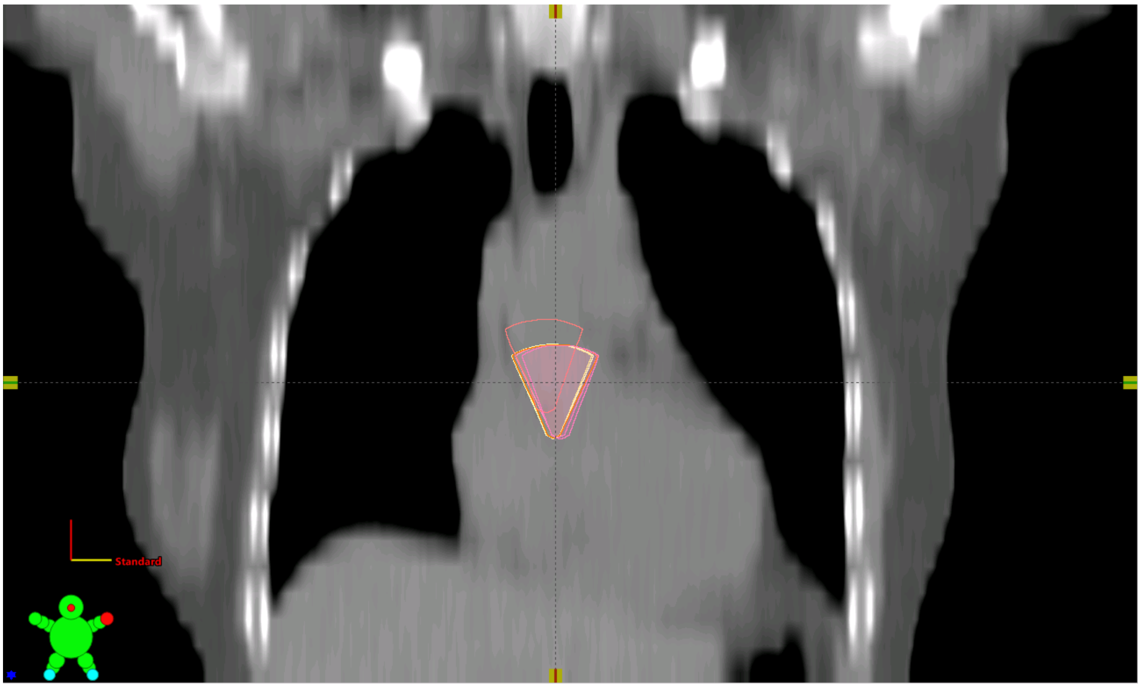

Figure S5: Contour delineations for the aortic valve – coronary plane.

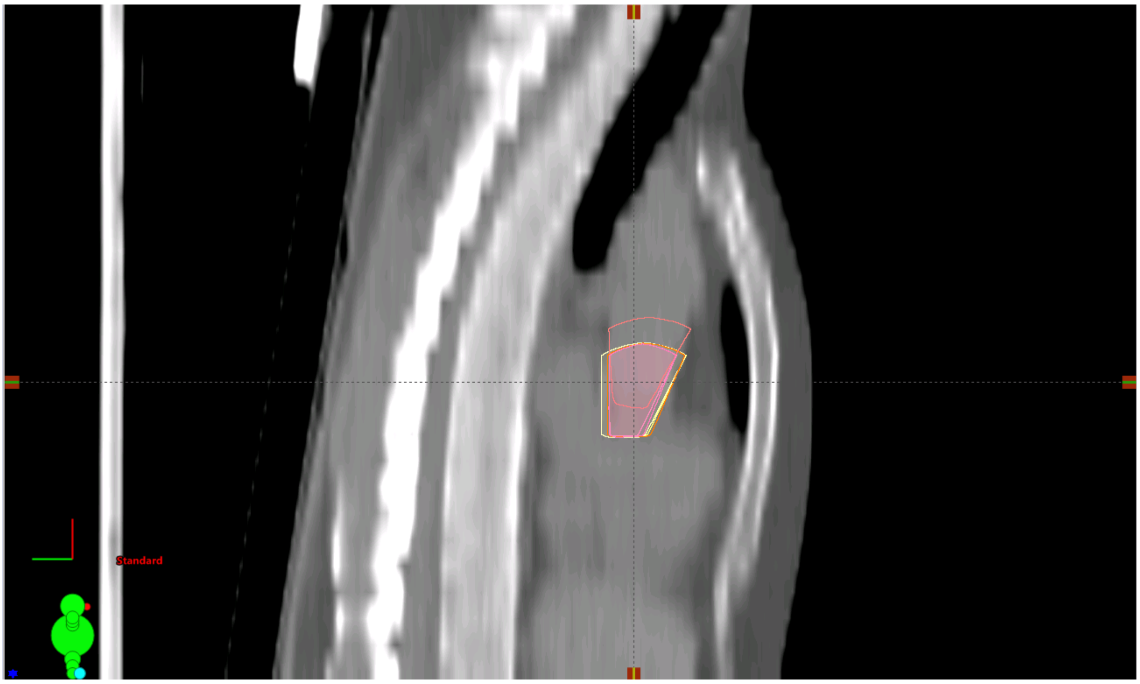

Figure S6: Contour delineations for the aortic valve – sagittal plane.

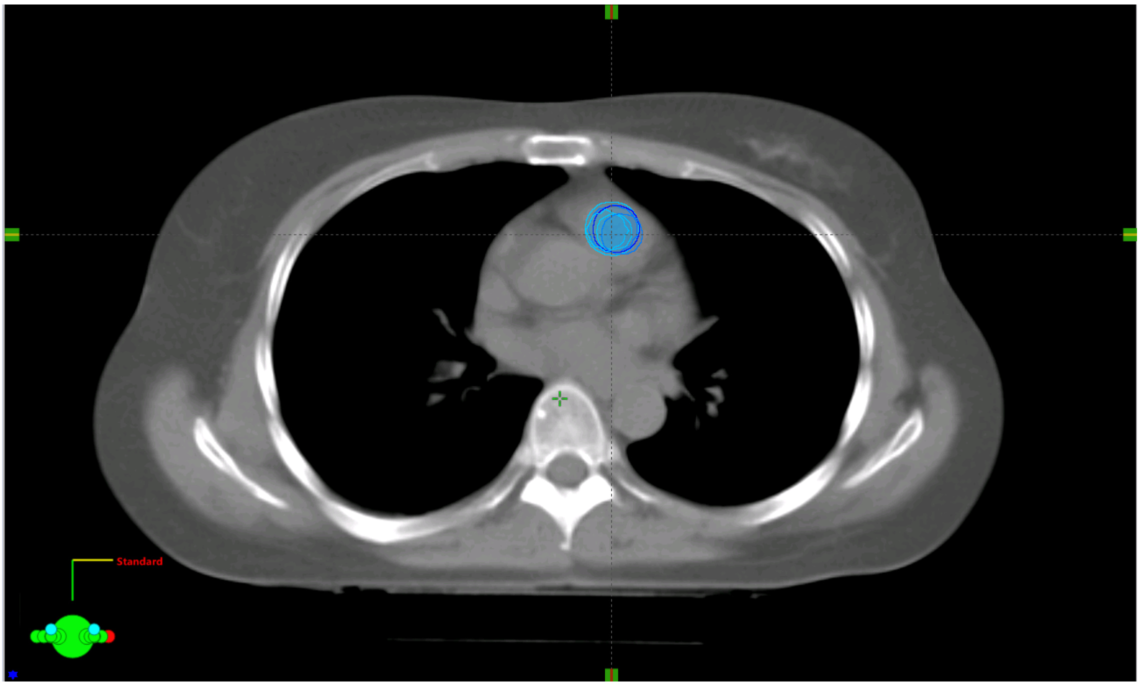

Figure S7: Contour delineations for the pulmonary valve – axial plane.

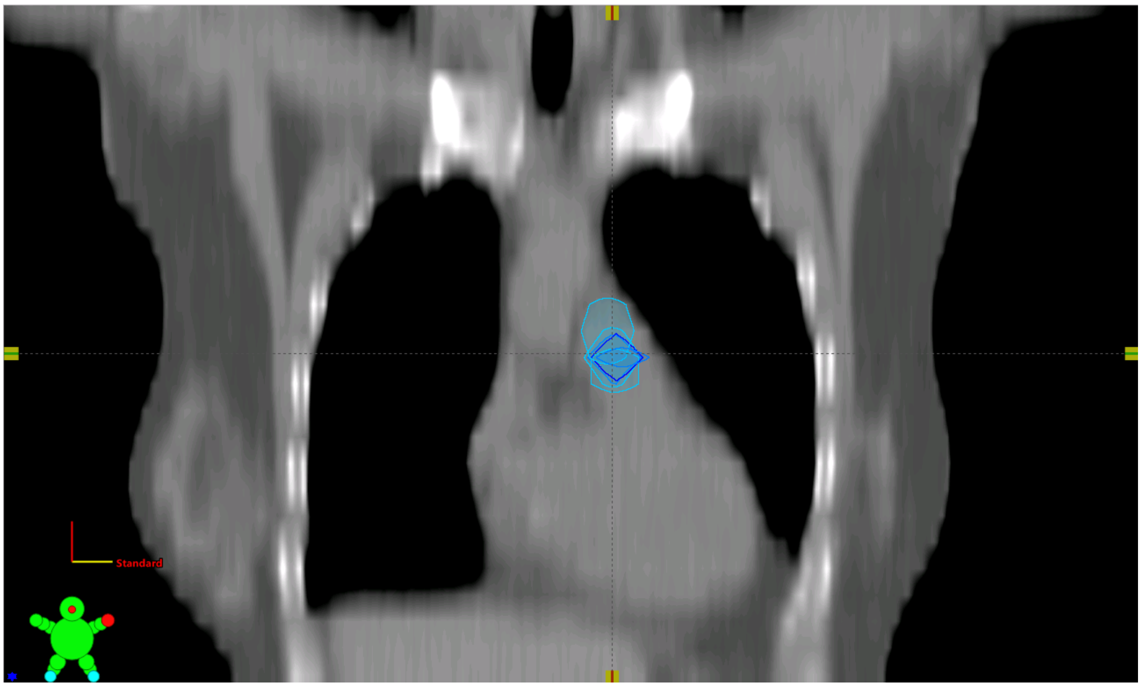

Figure S8: Contour delineations for the pulmonary valve – coronary plane.

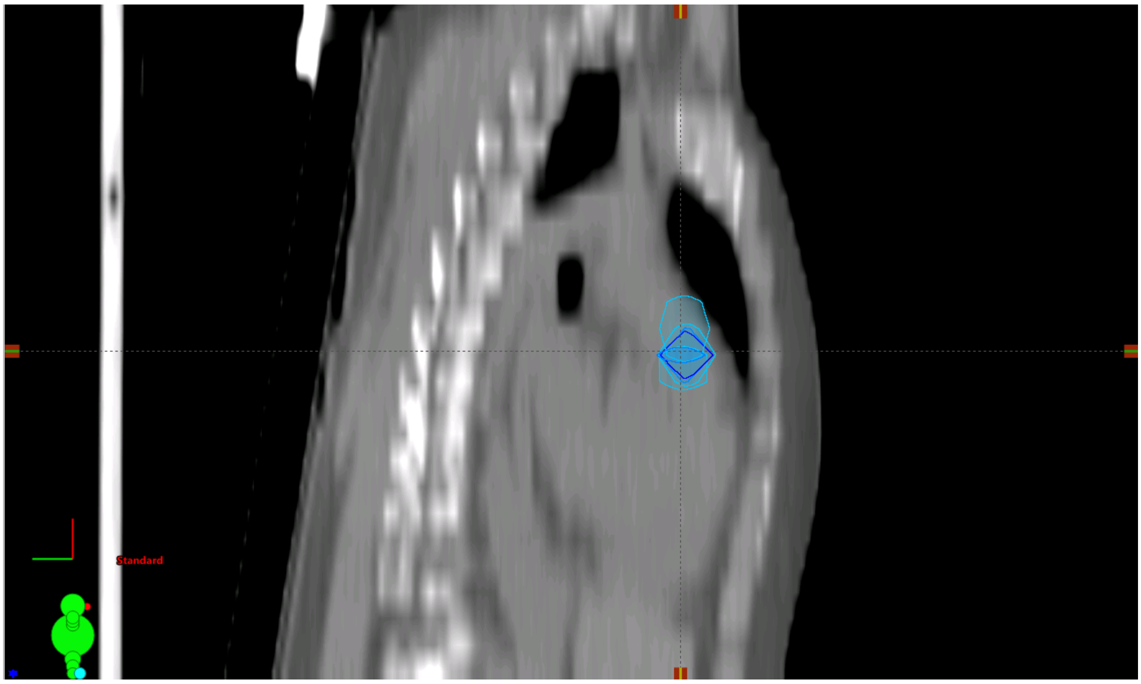

Figure S9: Contour delineations for the pulmonary valve – sagittal plane.

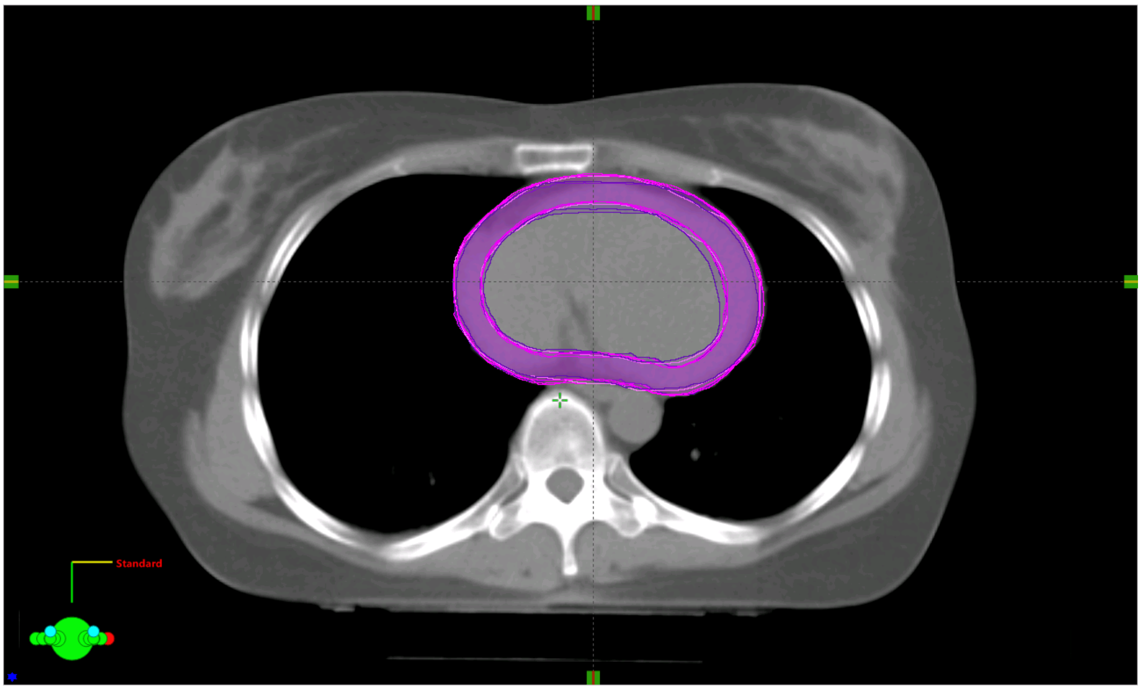

Figure S10: Contour delineations for the myocardium – axial plane.

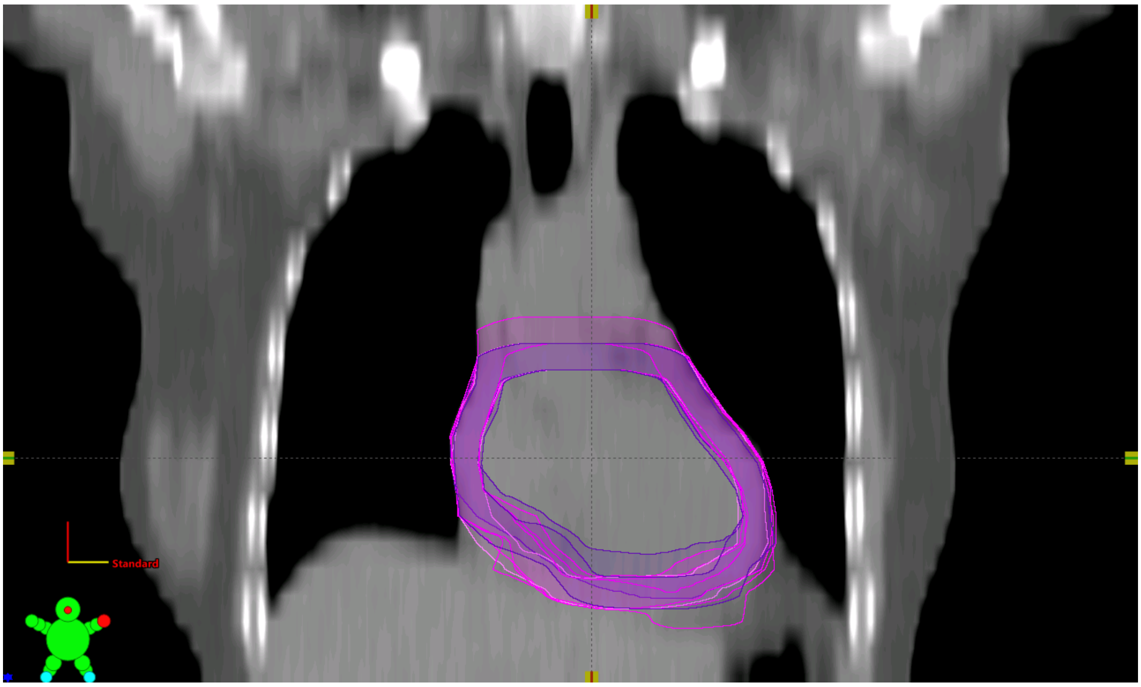

Figure S11: Contour delineations for the myocardiumt – coronary plane.

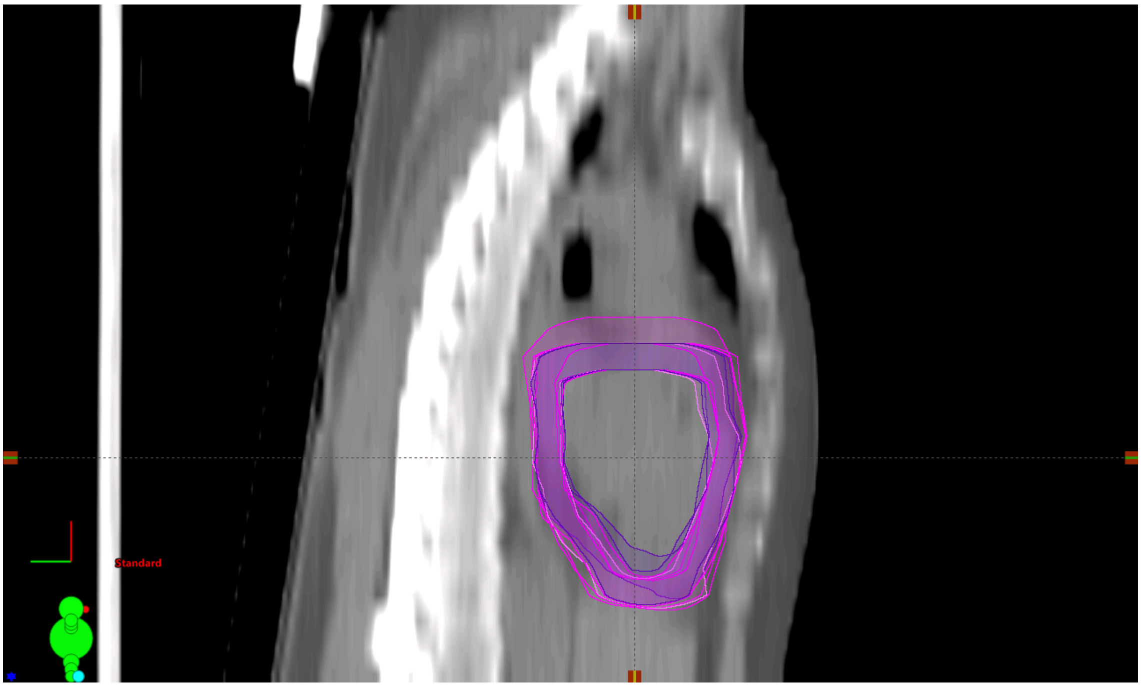

Figure S12: Contour delineations for the myocardium – sagittal plane.

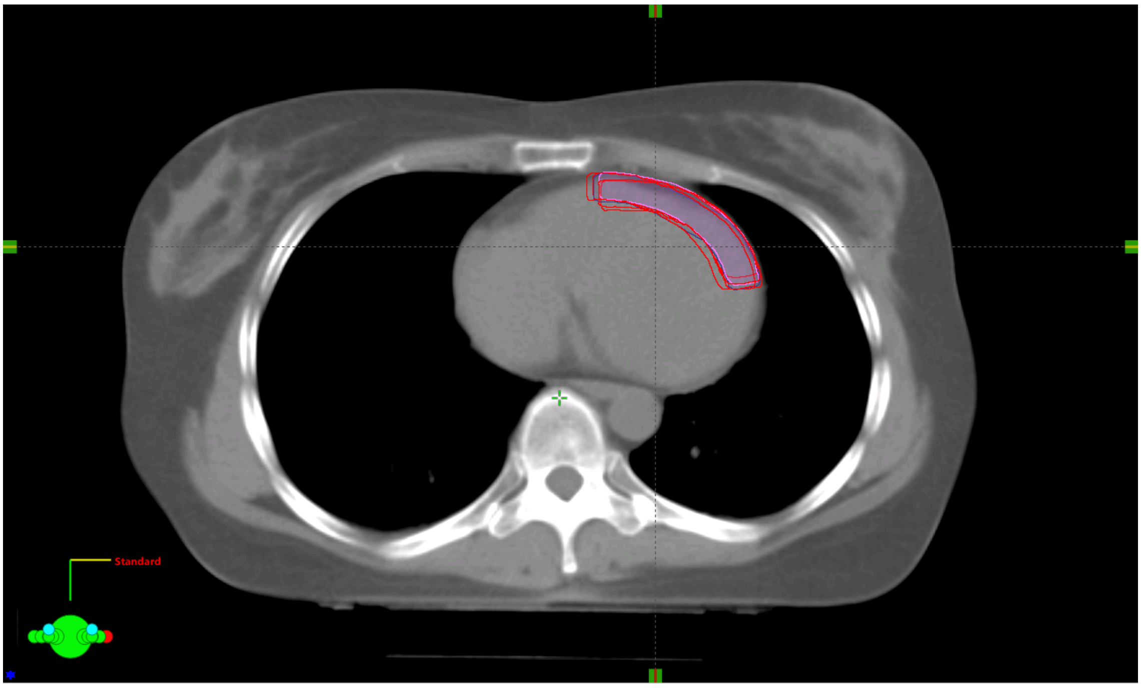

Figure S13: Contour delineations for the left anterior myocardium – axial plane.

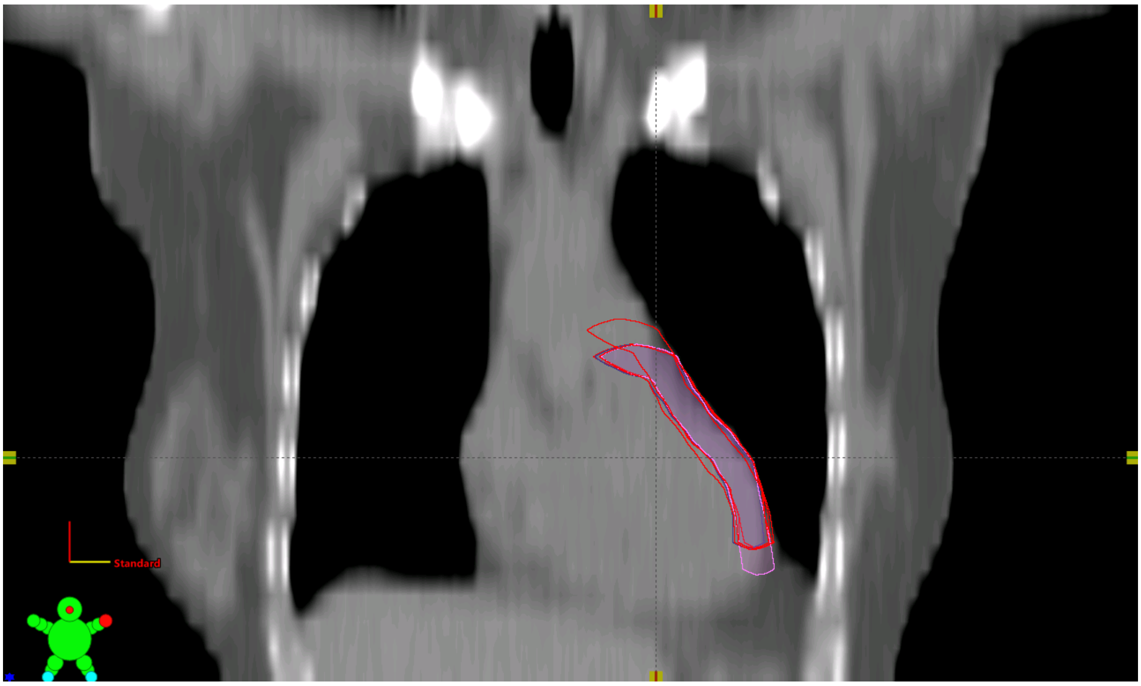

Figure S14: Contour delineations for the left anterior myocardium – coronary plane.

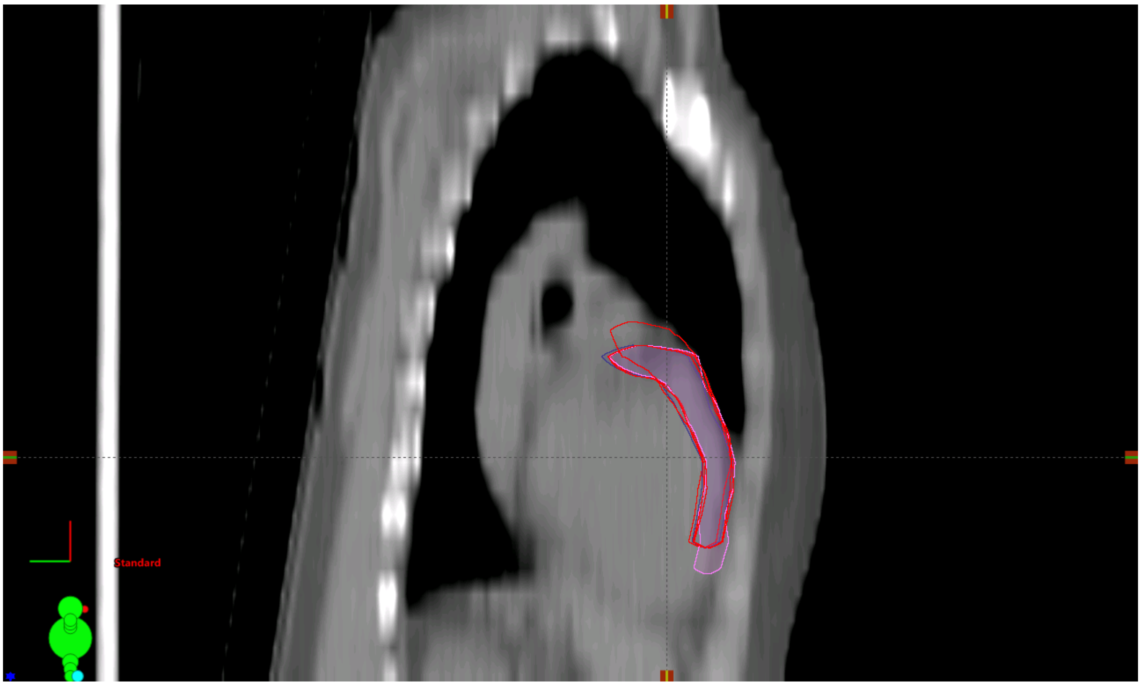

Figure S15: Contour delineations for the left anterior myocardium – sagittal plane.

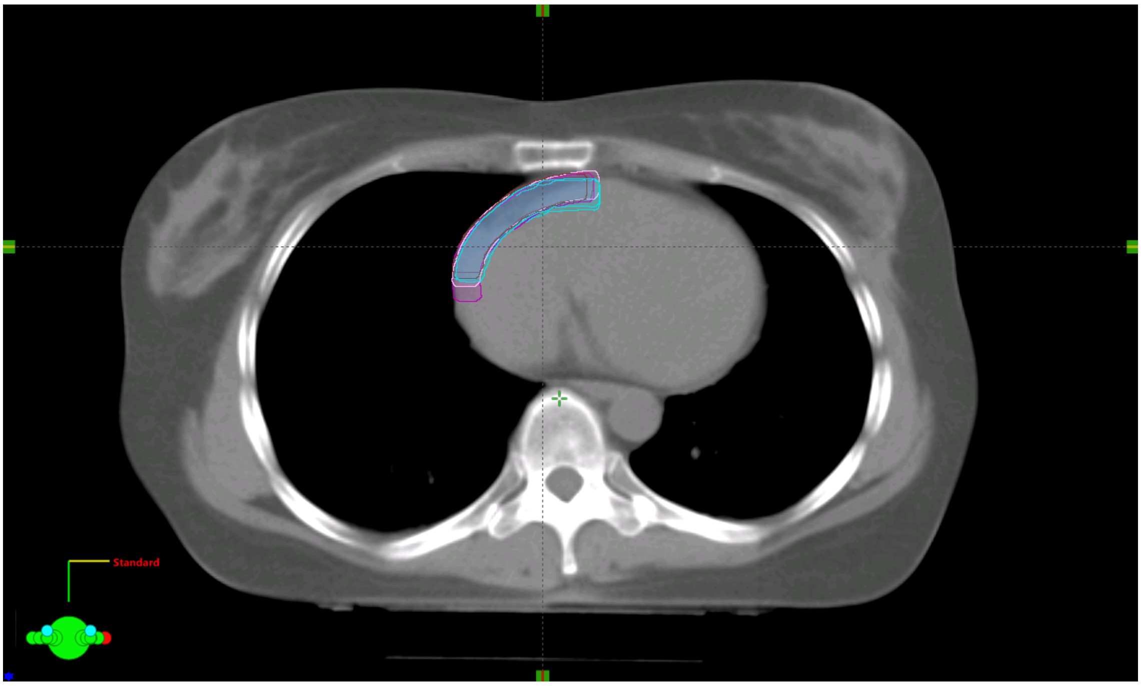

Figure S16: Contour delineations for the right anterior myocardium – axial plane.

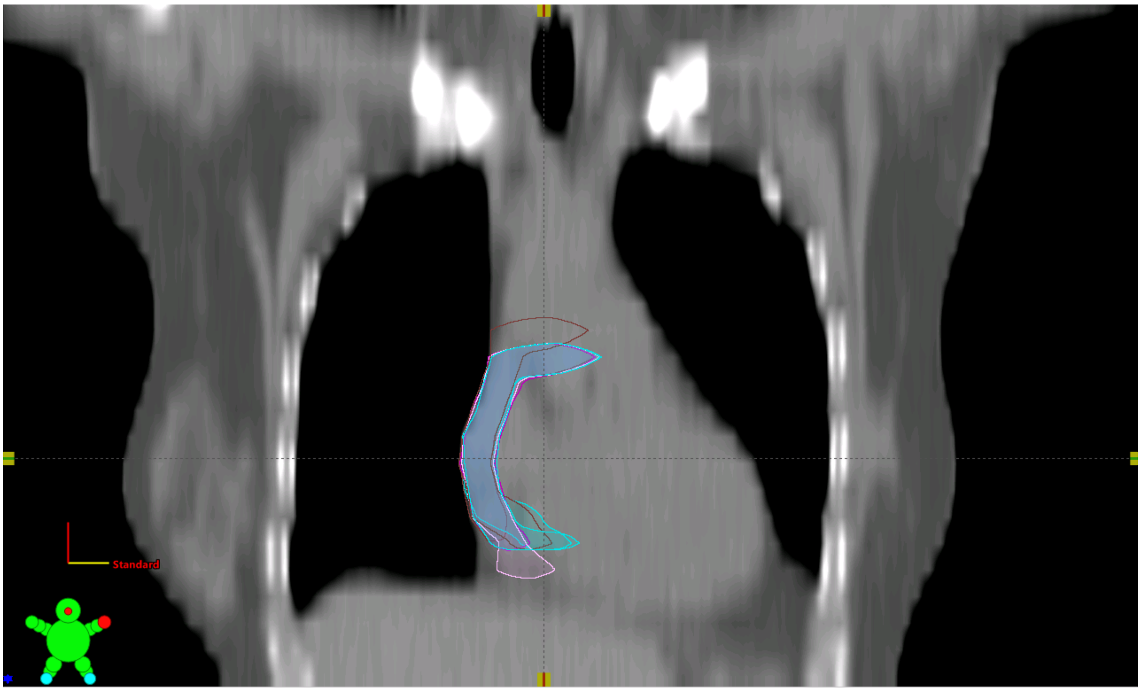

Figure S17: Contour delineations for the right anterior myocardium – coronary plane.

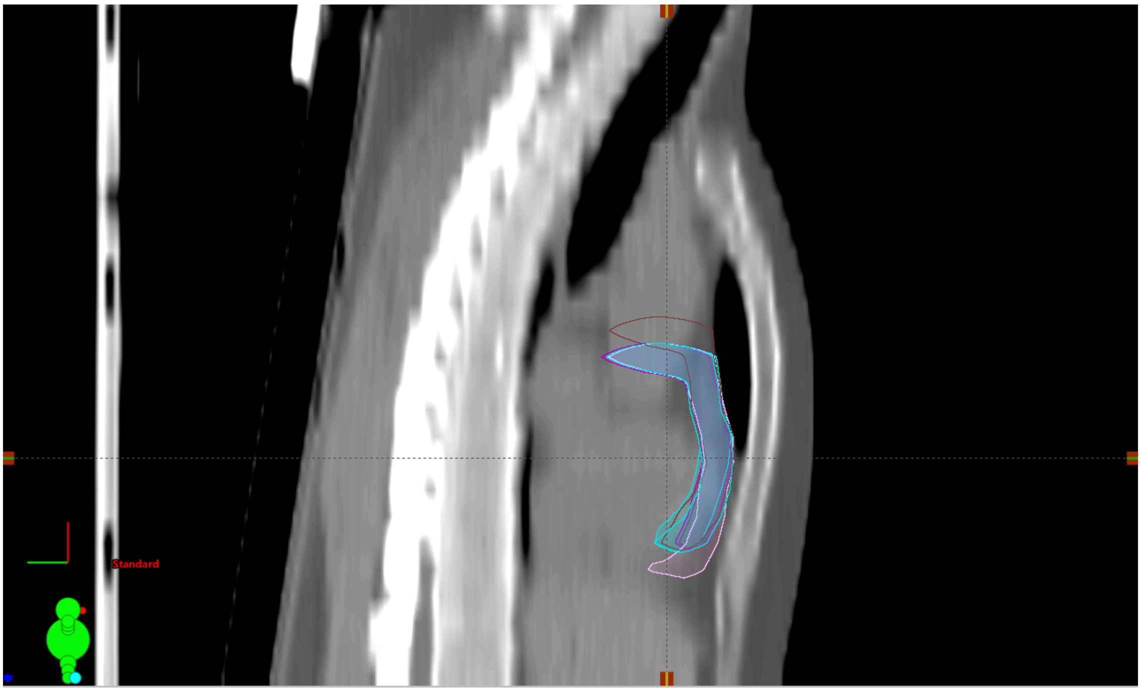

Figure S18: Contour delineations for the right anterior myocardium – sagittal plane.

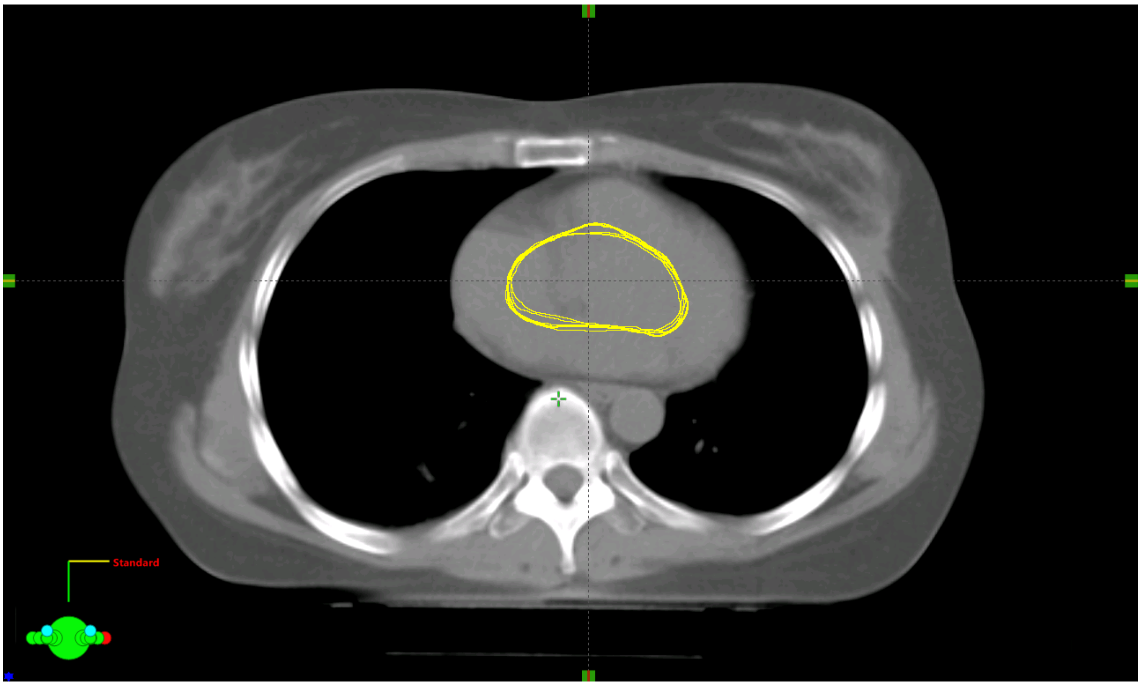

Figure S19: Contour delineations for the deep structures – axial plane.

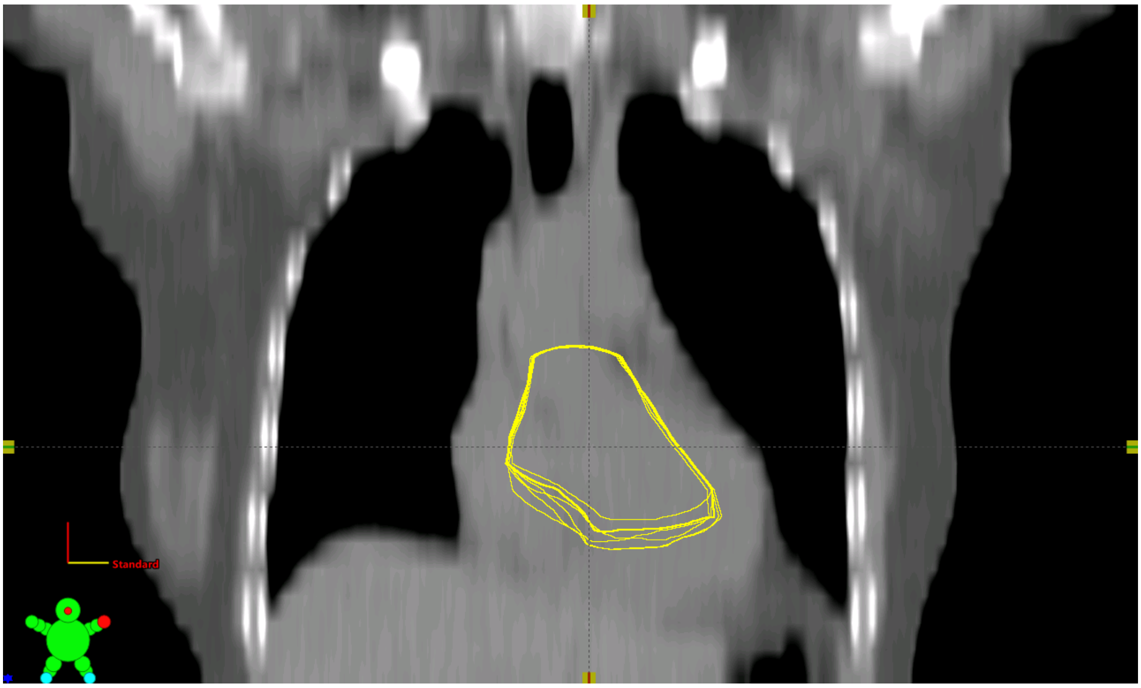

Figure S20: Contour delineations for the deep structures – coronary plane.

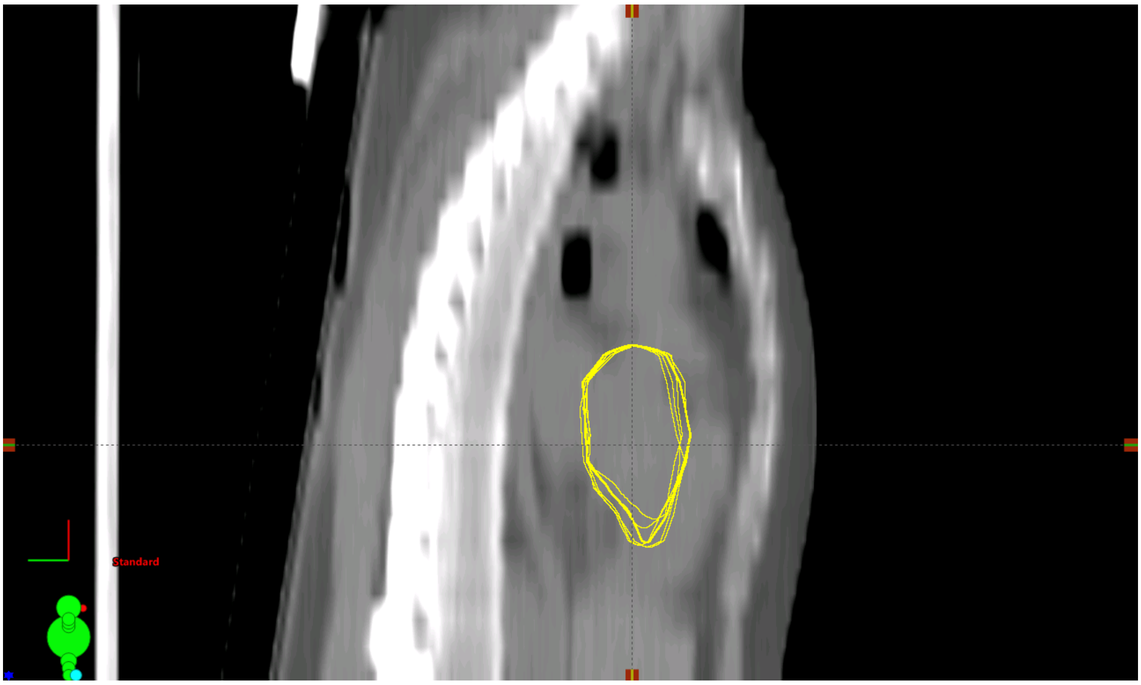

Figure S21: Contour delineations for the deep structures – sagittal plane.

2.2 Structure volume and absorbed dose

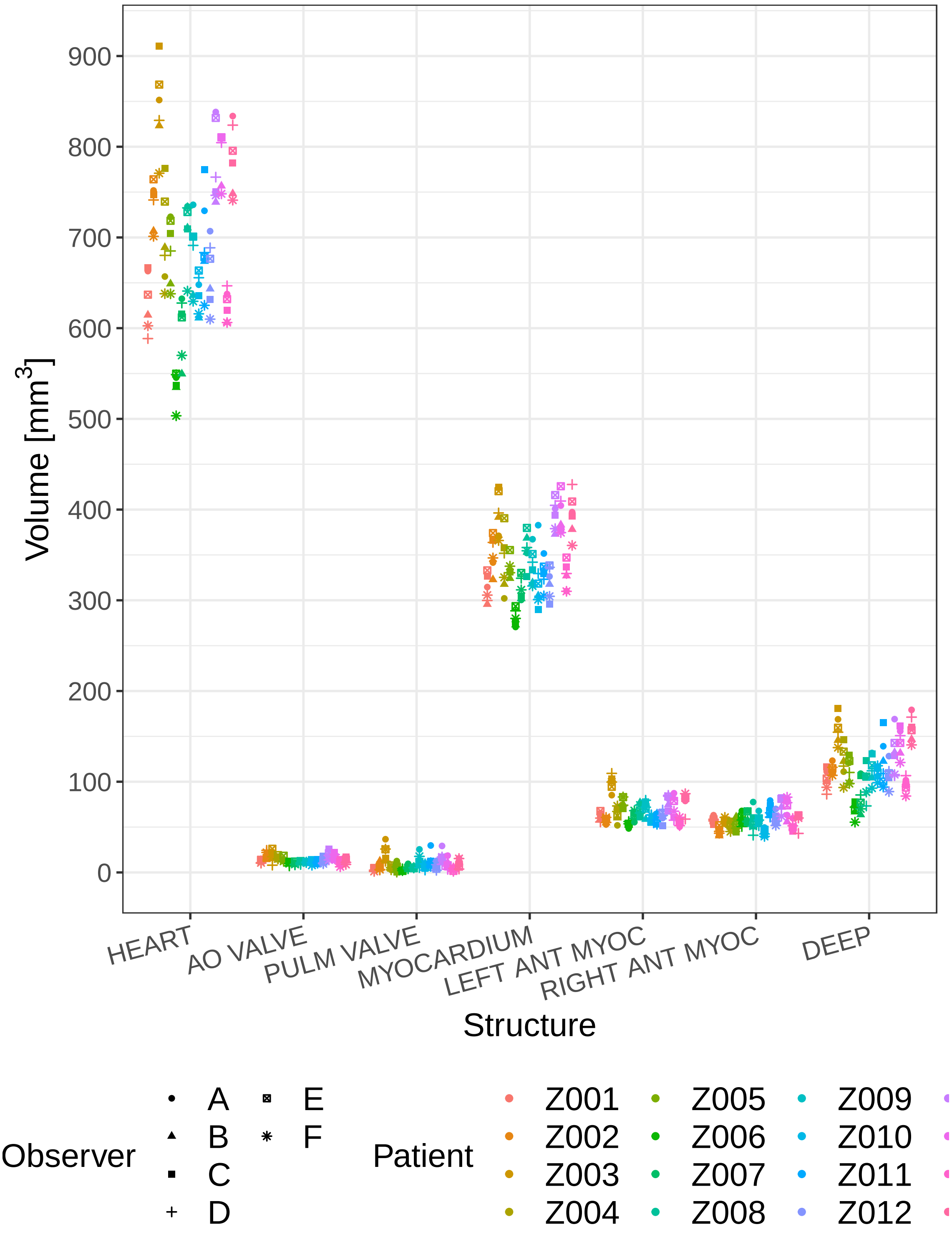

Figure S22: Structure volume for all 16 patients, all 7 heart atlas structures and all 6 observers.

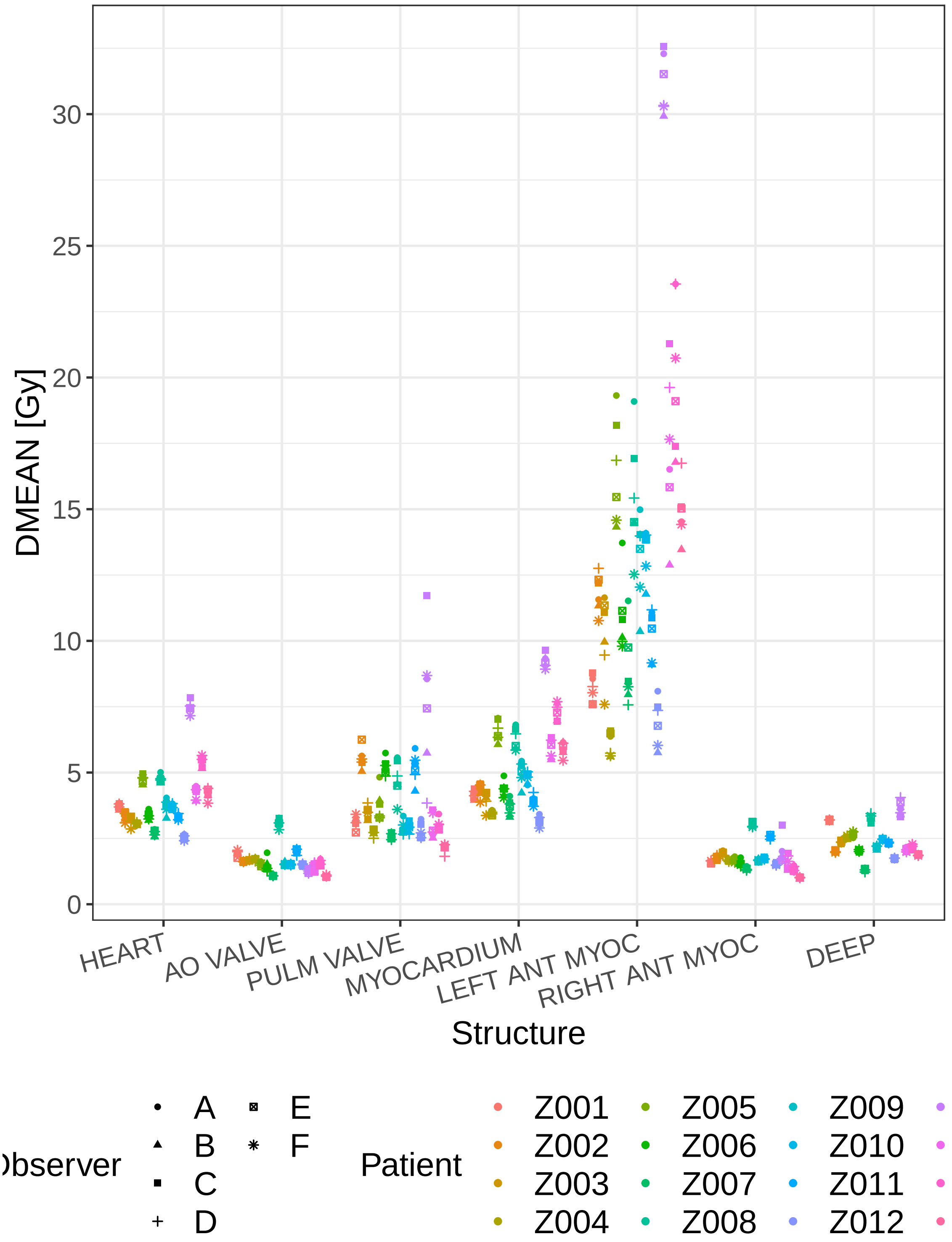

Figure S23: Mean absorbed dose (DMEAN) for all 16 patients, all 7 heart atlas structures and all 6 observers. Planned with a prescription dose of 50 Gy and boost of 10 Gy.

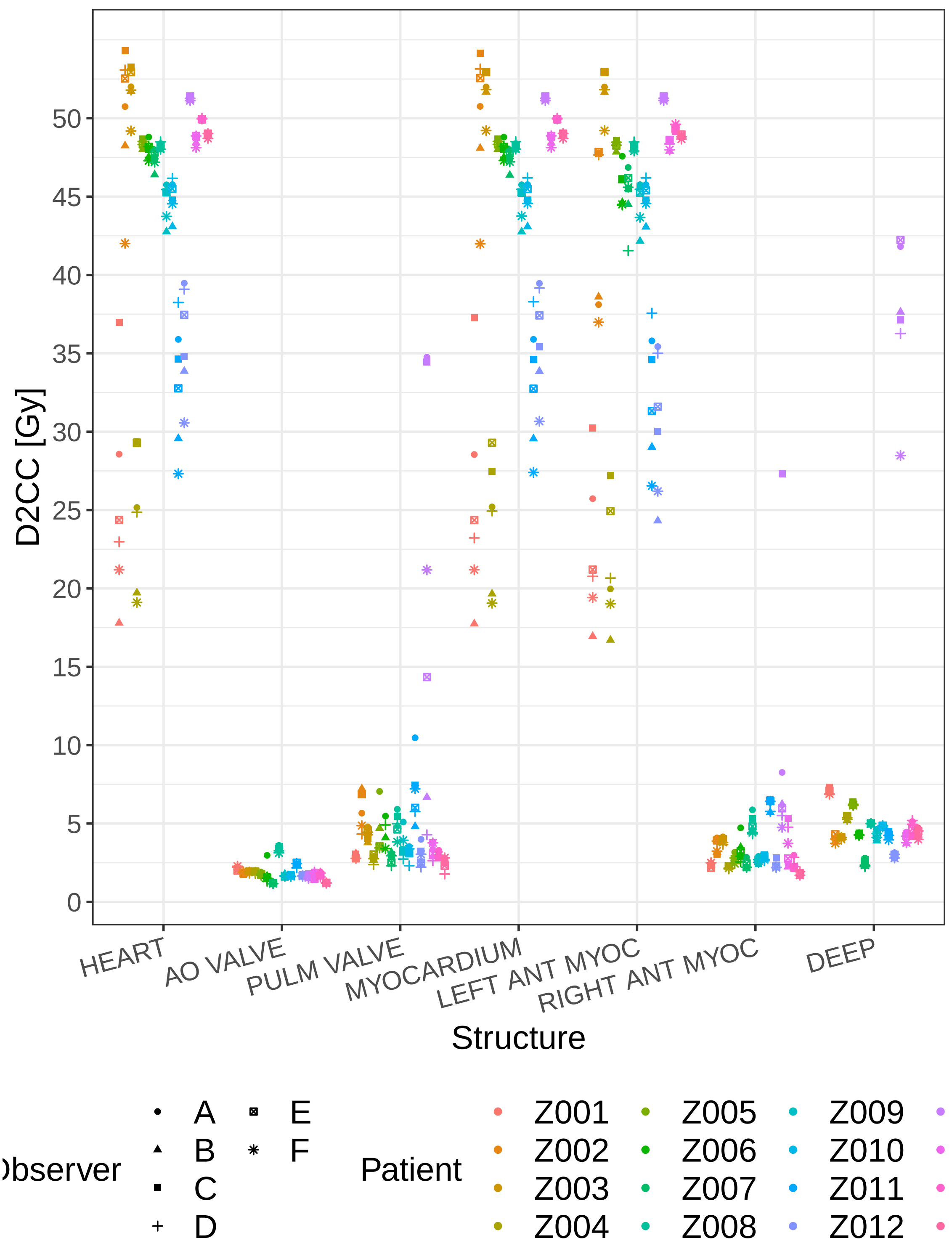

Figure S24: Extreme absorbed dose (D2CC) for all 16 patients, all 7 heart atlas structures and all 6 observers. Planned with a prescription dose of 50 Gy and boost of 10 Gy.

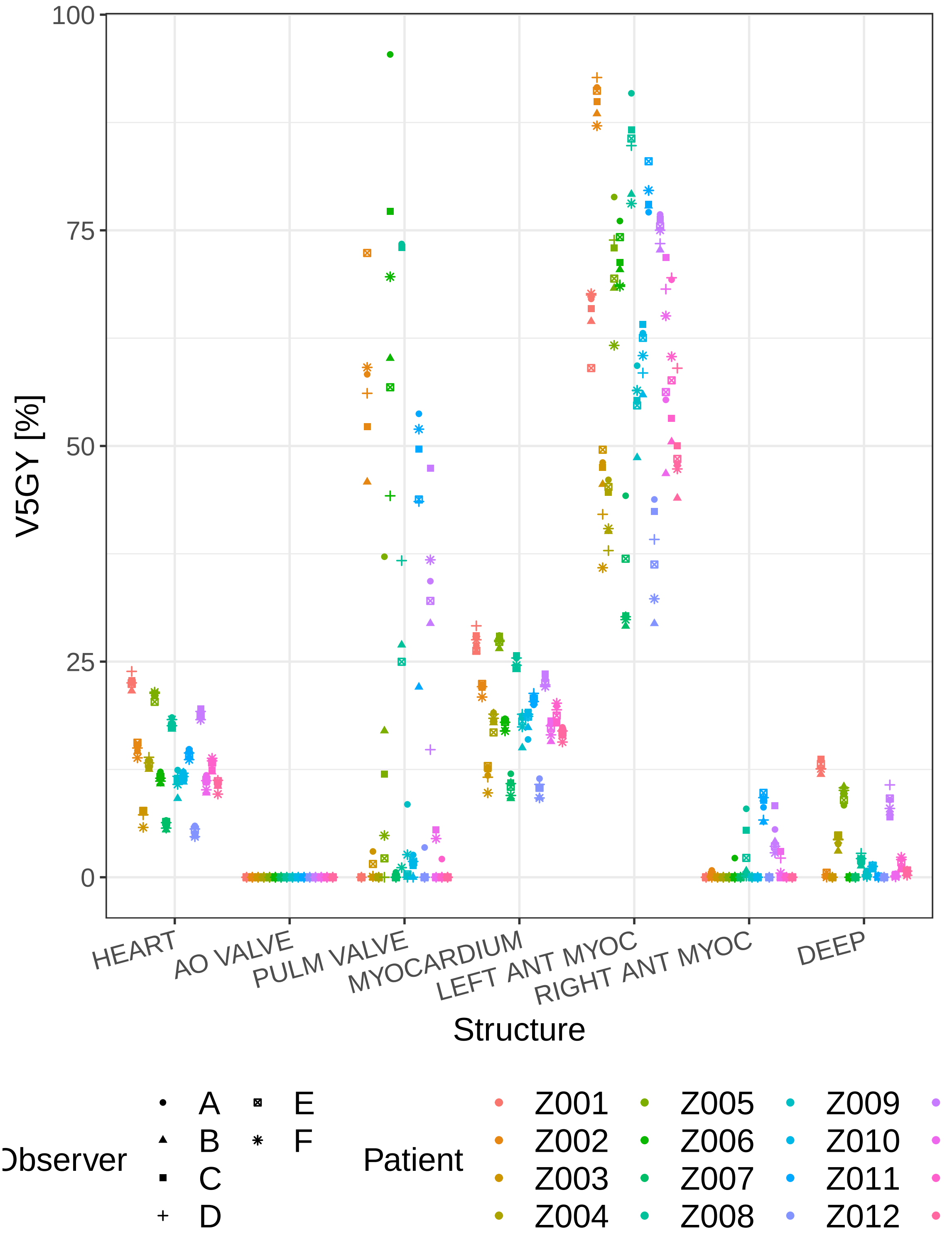

Figure S25: Relative volume with aborbed dose of at least 5 Gy (V5GY) for all 16 patients, all 7 heart atlas structures and all 6 observers. Planned with a prescription dose of 50 Gy and boost of 10 Gy.

References

Babalola, K., Patenaude, B., Aljabar, P., Schnabel, J., Kennedy, D., Crum, W., ... Rueckert, D. (2009). An evaluation of four automatic methods of segmenting the subcortical structures in the brain. *NeuroImage*, *47*, 1435-47.

Bürkner, P.-C. (2017). brms: An R package for Bayesian multilevel models using Stan. *J Stat Soft*, *80*(1), 1–28.

Dice, L. R. (1945). Measures of the amount of ecologic association between species. *Ecology*, *26*, 297–302.

Fotina, I., Lütgendorf-Caucig, C., Stock, M., Pötter, R., & Georg, D. (2012). Critical discussion of evaluation parameters for inter-observer variability in target definition for radiation therapy. *Strahlenther Onkol*, *188*, 160-167.

Hanna, G., Hounsell, A., & O'Sullivan, J. (2010). Geometrical Analysis of Radiotherapy Target Volume Delineation: a Systematic Review of Reported Comparison Methods. *Clin Oncol*, *22*, 515-525.

Jaccard, P. (1912). The distribution of flora in the alpine zone. *New Phytologist*, *11*, 37–50.

Jacobson, A., & Panozzo, D. (2021). libigl: A simple C++ geometry processing library [Computer software]. URL https://libigl.github.io/

Nürnberg, R. (2013). *Calculating the volume and centroid of a polyhedron in 3d* (Tech. Rep.). London, UK: Imperial College London. URL http://wwwf.imperial.ac.uk/~rn/centroid.pdf

R Core Team. (2021). R: A Language and Environment for Statistical Computing [Computer software manual]. Vienna, Austria. URL https://www.r-project.org/

Revelle, W. (2020). psych: Procedures for psychological, psychometric, and personality research [Computer software]. URL https://CRAN.R-project.org/package=psych (R package version 2.0.12)

Shrout, P. E., & Fleiss, J. L. (1979). Intraclass correlation: Uses in assessing rater reliability. *Psychological Bulletin*, *86*, 420–428.

Wollschlaeger, D., & Karle, H. (2020). DVHmetrics: Analyze dose-volume histograms and check constraints [Computer software]. URL https://CRAN.R-project.org/package=DVHmetrics (R package version 0.3.10)
